# The look of a leader

**DOI:** 10.1371/journal.pone.0320836

**Published:** 2025-04-03

**Authors:** Katherine Qianwen Sun, Michael L. Slepian, Modupe Akinola

**Affiliations:** 1 Anderson School of Management, University of California, Los Angeles, Los Angeles, California, United States of America; 2 Columbia Business School, Columbia University, New York, New York, United States of America; Australian Catholic University, AUSTRALIA

## Abstract

Women and minorities are underrepresented in top leadership roles. Besides “supply-side” explanations that focus on the applicant pool, we offer a novel “demand-side” explanation through perceptual imprinting. Using the reverse correlation method, we found that people’s visual templates of leaders are perceptually imprinted by White male leaders. Across 15 studies (N = 3929), we examine what people expect leaders to look like. As demonstrated by the reverse correlation method, compared to followers, people expected leaders to look more White and male. Across all social categories, people also expected leaders to look more dominant, competent, and powerful. But to look like a leader, Black and female leaders also needed to look likable. Additional differences were observed by participant gender. Thus, in addition to having biased perceptual representations of leaders, people also have different perceptual standards for different social groups, as expressed by different expectations for how leaders of different races and genders should look. (151 words).

## Introduction

At the highest levels of leadership, gender and racial inequity is vastly pronounced, with women and minorities being markedly underrepresented in leadership roles. Recent estimates highlight that fewer than 9% of Fortune 500 CEOs are women [1], and Black CEOs comprise only 1% of Fortune 500 board seats [2]. Gender and racial bias have been found to impact who is given promotions, assigned to board seats, and judged to have more power and status, often favoring White males [3,4].

Explanations for the underrepresentation of women and minorities in leadership often center around bias—namely, the differential treatment and perceptions of women and minorities, relative to White males, in the workforce (i.e., “demand-side” theories), or on the limited pipeline for women and minorities to fill top positions (i.e., “supply-side” theories; [5]). We offer a novel “demand-side” explanation for the underrepresentation of women and minorities in leadership positions: *perceptual imprinting*.

We propose that people’s visual templates for how leaders look have become skewed through constant exposure to a disproportionate number of White males in leadership positions, forming a perceptual imprint of leaders as White males. Given organizations often interview their candidates face-to-face, there exists no equivalent of the “blind audition” used by many orchestras, where a musician performs behind a screen (thus hiding visual qualities of what the musician looks like, including critically, gender and race). Aside from the many cognitive associations (stereotypes) people hold of different social groups, we lack knowledge of whether prescriptive gender and racial societal norms pervade people’s expectations of what a leader should look like at the *perceptual level*. In other words, is there bias in people’s internalized visual representations of leaders?

Prior research has shown that first impressions of leadership are largely affected by rapid judgments of faces [6–9]. Even from very brief exposure to a face (within 100 milliseconds), people generate complex social inferences about that person’s character [10,11], often at the expense of other more valid and diagnostic cues of leadership [12,13]. While prior work examined the facial features that prompt judgments of leadership [14–16], the current research reverses the causal arrow, asking whether thinking of a leader brings to mind specific facial features. This approach investigates the foundations of leadership biases at a perceptual level. We also examine the extent to which perceptions are static or dynamic, depending on target race and gender. In other words, moving beyond the social category imagined when thinking of the face of a leader, we ask what happens when the social category is itself manipulated? For example, if people associate leaders with male, what does this mean for how people imagine female leaders? And what about Black leaders? The current work explores these novel questions.

Using a reverse correlation perceptual paradigm, we show that despite *constraining the starting point* of an imagined face to be perceptually gender-ambiguous and race-ambiguous, for that face to look like a leader, relative to a follower, it needs to look more White and male (Study 1). This finding is consistent with a literature showing that people associate leadership with the male social category (e.g., “think leader, think male”; [17]), and is also consistent with literature showing that people associate leadership with the White social category [18,19]. Importantly, these two traditions have yet to intersect, such that the former has yet to examine the imagined race (of the imagined male leader), nor has the “think leader, think White” literature considered the imagined gender (of the imagined White leader). The current Study 1 was uniquely capable of measuring the imagined race and gender of the face people picture when thinking of a leader.

After testing for this intersection in Study 1, the current work moves beyond it (and other standard reverse-correlation studies) in two major ways. First, across Studies 2-5, we experimentally constrain the face space of the imagined face. For example, across Studies 2 and 3 we can ask whether target gender moderates any effect of imagining leaders as looking more White than followers. Likewise, across Studies 4 and 5, we ask whether target race moderates any effect of imagining leaders as looking more male than followers. Second, in addition to having other participants rate the perceived gender and race of the faces, we ask participants to perceive a set of highly relevant traits (e.g., dominance, competence, power, warmth, likeability, happiness). Thus, if constraining the face space of imagined faces influences what imagined leaders look like, we can better understand the nature of these social categorical influences by examining the psychological traits attributed to the face. We examine traits highly studied in the face perception literature as well as those highly relevant to leadership.

### Gender

One simple manifestation of a biased visual representation of leaders is that when a person imagines a leader, they image a male. But this bias might manifest itself in several additional ways beyond the social category imagined. For example, what do people imagine a female leader looks like? If people visually associate leadership with the male social category, people might even expect their women leaders to look more dominant.

Importantly, there are two broad dimensions by which people consensually perceive faces: dominance (low brows, more angular face, downturned mouth) and trustworthiness/ positivity (upturned eyebrows, less prominent brow, rounder face, upturned mouth; [20]). These perceptual features are also gendered, whereby the first set of features is typical of males faces, whereas the second set of features is typical of female faces [21]. And even conceptually, the constellation of traits that dominance sits in (e.g., agentic, competent, powerful) overlaps with what is considered masculine, and the constellation of traits that trustworthiness/positivity sits in (e.g., warm, friendly, communal) overlaps with what is considered feminine [22].

Accordingly, if people have existing visual templates for how a leader should look, then when they are asked to imagine the face of a leader with no social category constraints, they might imagine a person who looks relatively White and male and who looks dominant rather than positive. But what about when asked to imagine a female leader? Would she look dominant rather than positive? Or might she need to look dominant *and* positive?

In the current work, we use a perceptual paradigm to test these questions, whereby we present to participants a perceptually gender-ambiguous and race-ambiguous face with random visual noise, and ask participants to consider whether that face looks leader-like (and in a separate study, follower-like). If by chance the random visual noise makes the face look more like a male, for example, then people may be more likely to say it looks like a leader (or, does not look like a follower).

If people hold *gendered* expectations of what leaders look like, compared to imagining a follower, people may, 1) imagine a leader looks more White and male. But also, 2) for a female target face to look like a leader, that target may also need to look masculine (i.e., more dominant). And by the same logic, 3) for a Black target face to look like a leader, relative to a follower, it may also need to look masculine (i.e., more dominant).

### Race

Another potential manifestation of a biased visual representation would be to imagine a leader as looking more White than what people imagine a follower looks like. This would be consistent with the finding that people associate leadership with the White social category [18,19].

Moreover, a unique feature of our designs is that they allow us to compare the magnitude of gender effects to race effects. For example, if when asked to imagine the face of a leader, compared to a follower, a participant might imagine a relatively White face, but one that is *very clearly male*, which would suggest a larger gender effect. If another participant imagined a face that was relatively male-looking, but *very clearly White*, that would suggest a larger race effect.

### Gender and race intersections

In addition to being able to compare the magnitude of gender and race effects, our designs also enable us to consider the intersection between these two variables. For example, with our Study 1 design we can ask: Is the gender effect larger than the race effect? Additionally, in our follow-up studies, we can ask: How does any gender effect differ by race? For example, when envisioning the face of a Black leader that face may appear less dominant and more positive than the imagined face of a White leader. This would be consistent with the proposed benefit of “disarming” cues for Black men [23].

In addition to experimentally constraining the imagined faces, by also measuring additional features of the imagined face, we consider whether standards (to look leader-like) will shift with the social category of the face. This allows for testing intersectionality theories, such that we can ask—across different social categories—1) who needs to look dominant to look more like a leader, 2) who needs to look dominant and positive, and 3) who needs to look positive but not too dominant.

## The current work

Using a reverse correlation paradigm, we demonstrate that people have highly nuanced perceptual representations for how leaders’ and followers’ faces should look, which dynamically update according to the race and/or gender of the target in question. Our results indicate that people imagine both White and Black *leaders* to look more masculine compared to what they imagine White and Black *followers* look like. Further, imagined male and female *leaders* look more White than imagined male and female *followers*. Overall, when participants are simply asked to imagine a leader, they imagine someone who looks relatively more White and male than when they imagine a follower. Notably, exceptions were observed for female leaders and Black leaders. Compared to followers, female leaders were perceived as more female, and Black leaders were perceived as more Black, and, as discussed below, other notable differences emerged on trait ratings. The pre-registration report for the current studies can be found on AsPredicted (https://aspredicted.org/p74t-b5sd.pdf). All hypotheses, experimental conditions, and planned analyses were specified in advance. The dataset and analysis code used in this study are publicly available on the Open Science Framework (OSF) at https://osf.io/n8upr/. All materials, including survey instruments and stimuli, can also be accessed through this repository.

## Study 1: What does a leader look like?

The goal of Study 1 was to examine people’s visual templates for what leaders and followers look like. Specifically, we presented to participants a perceptually race-ambiguous and gender-ambiguous base face, on which visual noise was superimposed. Thus, Study 1 allowed us to infer the perceptual imprint of leaders, broadly speaking.

### Participants and methods

#### Design.

The perceptual reverse-correlation task [24] tests how people visualize faces along any dimension. Studies 1a and b (image-generation) exposed participants to a race-ambiguous and gender-ambiguous face, superimposed with random visual noise. Next to this face, was the same face, superimposed with the inverse of that noise. This created two slightly different-looking randomly-generated versions of the same face, and Study 1a participants’ task was simply to choose the face that looked more like a leader [“Which image looks more like a leader, boss, or manager (i.e., someone who is in charge of other people)?”], whereas Study 1b participants chose the face that looked more like a follower [Which image looks more like a subordinate or follower (i.e., someone who does what others tell them to do)?”]. Participants completed 300 such trials, creating many different visual variants (using the default from the “rcicr” package). By averaging over the random visual noise across all selections [25], we could produce the average image of what made the faces appear more like a leader (Study 1a), or a follower (Study 1b).

In Study 1c (image-rating), a separate group of participants rated the leader and follower visualizations created by Study 1a and 1b participants on different dimensions related to leadership.

#### Participants and procedure.

We sought 250 participants from CloudResearch’s Connect platform for each study. Participants were recruited from this pool because they are representative and reliable [26], and this sample size was based on a recent simulation for reducing the Type I error rate in the reverse correlation method [27]. Since the study design required participants to follow detailed instructions, including downloading materials to their desktop, it was crucial that they adhered to each step carefully. To ensure that participants were engaged and following the instructions, a free-response question was included at the end of the survey. This question, which asked participants to write at least one sentence about a recent conversation they had, served as an instruction-following check. By including this step, we aimed to verify that participants were attentive and reliable in completing the study tasks, ensuring the integrity of the collected data. Any participants who failed to provide an appropriate response were excluded from the analysis. The same exclusion criterion applied to all image selecting studies. When a participant did not submit their study code (for compensation), this allowed an additional participant to take part; 252 participants completed Study 1a (*M = *37.09 years, *SD* =  11.60 years, 117 female, 134 male and 1 non-binary participants; 65.87% White, 15.08% Black, 7.14% Hispanic, 8.73% Asian, and 3.17% other racial background participants), and 251 participants completed Study 1b (*M = *39.97 years, *SD* =  11.78 years, 126 female, 125 male participants; 71.71% White, 9.16% Black, 8.76% Hispanic, 6.37% Asian, and 3.98% other racial background participants).The studies were approved by a local Institutional Review Board.

#### Image-generation studies (Studies 1a and b).

**Fig 1:**
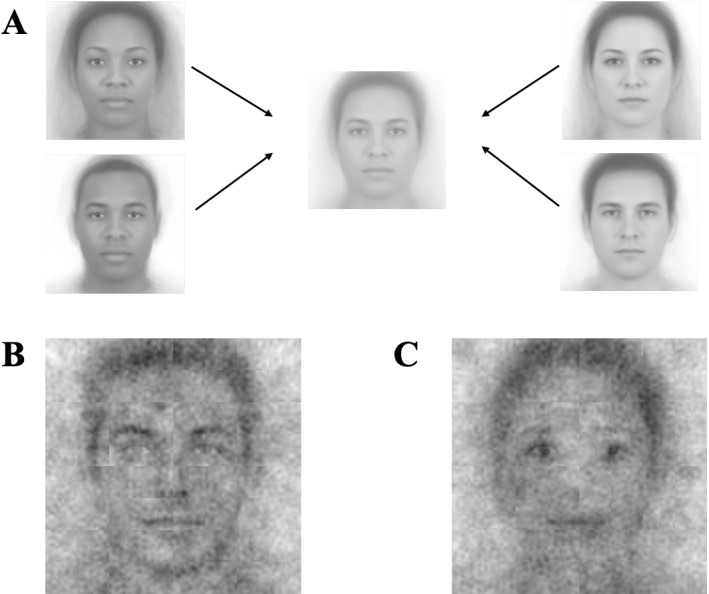
Visual composites for Study 1. Image attribution: “Ma, Correll, & Wittenbrink (2015). The Chicago Face Database: A Free Stimulus Set of Faces and Norming Data. Behavior Research Methods, 47, 1122-1135.” Reprinted from Chicago Face Database under a CC BY license, with permission from The University of Chicago Center for Decision Science, original copyright 2015. (A) Composite Image. (B) Visual composite of all leader selections. (C) Visual composite of all follower selections.

To create the base-images for Studies 1a and 1b, we created a composite face from 219 pictures from the Chicago Face Database [28]—53 Black female, 53 Black male, 56 White female, and 57 White male images. First, ambiguous mono-social categorical images were generated by blending the 50 + images from each social category. Later, the four blended images, one from each social category, were blended again to generate the gender and race ambiguous image. Importantly, a perfect blend of the four blends may not be perfectly race-ambiguous and gender-ambiguous, and so we pre-tested different blends to arrive at the blend that was rated by a separate group of participants to be ambiguous with respect to both gender and race (see Fig 1A; for the pre-test results, see SOM). Thus, if the resulting composite images appeared to have features of a gender or race, this would be driven by participants’ imagined representations of what leaders look like, as the base-face upon which the noise was superimposed was race-ambiguous and gender-ambiguous.

Per each Study 1a and 1b trial, we added random visual noise to the race-ambiguous and gender-ambiguous base face. Following Brown-Iannuzzi et al. [29,30], the noise-patterns on the base-images consisted of superimposed truncated 2-cycle sinusoid patches in all combinations of six orientations (0°, 30°, 60°, 90°, 120°, and 150°), five spatial scales (2, 4, 8, 16, and 32 patches per image), and two phases (0, π/2), with random contrasts.

The average visual noise-pattern of all *selected* images across all participants of a leader’s face from Study 1a and of a follower’s face from Study 1b are shown in Fig 1B and 1C.

#### Image-rating study (Studies 1c).

In Study 1c, participants rated the composite images generated from Studies 1a and 1b. A recent paper recommends obtaining 20–40 ratings per stimulus for reliable means [31]. We followed this recommendation, targeting a total sample size of 300 participants, with 30 participants rating the images on each of the 10 different ratings. The same attention check question was included at the end of the survey and the same exclusion criterion applied to all rating studies. We received 300 complete responses and after the exclusions, the final sample size was 293 CloudResearch Connect participants (*M = *37.91 years, *SD* =  12.12 years; 147 female and 146 male participants; 67.92% White, 13.99% Black, 8.19% Hispanic, 7.85% Asian, and 2.05% other racial background participants), yielding about 30 participants per each of the 10 ratings. Each group of participants only completed one rating, keeping Type I error rate uninflated [27].

A total of 10 dimensions were assessed in Study 1c. Two of the dimensions considered social category appearance, gender: (1) Definitely Male to (6) Definitely Female (6); race: (1) Definitely White American to (6) Definitely African American (6) and the remaining 8 dimensions were standard person perception judgments, capturing *positivity* (items include attractiveness, happiness, likability, trustworthiness, and warmth) and *dominance* (items include competence, dominance, and powerfulness) on similarly worded six-point scales, following prior face evaluation and leadership research [7,31,32]. These dimensions served as our key dependent variables. Each Study 1c participant was randomly assigned to rate both leader composite images and follower composite images on a single randomly selected dimension (of the ten dimensions).

Typically, reverse correlation paradigms generate a single composite image for the rating stage of the study. This approach, however, reduces statistical power. A design that yields more images for rating increases statistical power, and ensures any results are not particular to the single composite image generated. Accordingly, we created 13 leader composite images (from Study 1a) and 13 follower composite images (from Study 1b). One composite averaged over all selections (across all participants’ trials), one averaged over all male participants’ selections, one averaged over all female participants’ selections, and then all selections were randomly divided into ten pools, from which we generated 10 more composites per each random pool of leader selections (Study 1a) and follower selections (Study 1b).

Per each of the 26 composite images, participant answered the question, “How X does this person look?” X representing one random dimension selected of the 10 total dimensions.

### Results and discussion

Multilevel modeling was used in all studies, including random intercepts for the image and the person rating the image. The leader composite images were judged as appearing more like a male than the follower composite images, *b* =  −3.03, 95% CI =  [−3.17, − 2.88], *SE* =  0.08, *t*(687.00) =  −39.67, *p* <  0.001, and more White, *b* =  −1.21, 95% CI =  [−1.35, −1.06], *SE* =  0.07, *t*(737.00) =  −16.50, *p* <  0.001. The descriptive statistics of these measures can be found in Table 1.

**Table 1:** Descriptive statistics for the leader and follower ratings. Study 1c.

Category	Dimension	Condition	Mean^b^	SD
Perceived Demographics	gender^a^	leader	1.74	1.14
		follower	4.77	1.15
	race	leader	2.74	1.29
		follower	3.95	1.04
Perceived Dominance	competence^c^	leader	4.31	1.21
		follower	3.28	1.24
	dominance	leader	4.26	1.00
		follower	2.89	1.04
	powerfulness	leader	4.45	1.00
		follower	2.63	1.05
Perceived Positivity	attractiveness	leader	4.12	1.02
		follower	2.90	1.34
	happiness	leader	3.49	0.86
		follower	3.42	0.98
	likability	leader	3.78	1.11
		follower	3.41	1.23
	trustworthiness	leader	3.43	1.16
		follower	3.62	1.15
	warmth	leader	3.25	1.18
		follower	3.18	1.30

^a^Higher ratings in Gender indicate the image was more likely perceived as Female, and higher ratings in Race indicate the image was more likely perceived as African American.

^b^All ratings are on a 1–6 point scale.

For example, 1 means “Definitely MALE/ WHITE AMERICAN” and 6 means “Definitely FEMALE/ AFRICAN AMERICAN” in the perceived demographics ratings.

^c^In the other ratings, 1 means “Definitely INCOMPETENT/SUBMISSIVE/ POWERLESS/ UNATTRACTIVE/ UNHAPPY/ UNLIKABLE/ UNTRUSTWORTHY/ COLD” and 6 means “Definitely COMPETENT/ DOMINANT/ PWERFUL/ ATTRACTIVE/ HAPPY/ LIKABLE/ TRUSTWORTHY/ WARM”.

Relative to the follower composite images, the leader composite images received more favorable ratings for the items that represent dominance: more competent (*b* =  1.03, 95% CI =  [0.90, 1.16], *SE* =  0.07, *t*(737.00) =  15.20, *p* <  0.001), more dominant (*b* =  1.37, 95% CI =  [1.24, 1.50], *SE* =  0.07, *t*(737.00) =  20.62, *p* <  0.001), and more powerful (*b* =  1.82, 95% CI =  [1.68, 1.96], *SE* =  0.07, *t*(712.00) =  26.18, *p* <  0.001).

At the same time, relative to the follower composite images, the leader composite images received more favorable ratings for items that represent likability: more attractive (*b* =  1.22, 95% CI =  [1.10, 1.34], *SE* =  0.06, *t*(712.00) =  19.76, *p* <  0.001) and more likable (*b* =  0.37, 95% CI =  [0.24, 0.48], *SE* =  0.06, *t*(712.00) =  6.11, *p* <  0.001)—although they received less favorable rating for perceived trustworthiness (*b* = −0.19, 95% CI = [−0.34, −0.04], *SE* = 0.07, *t*(737) = −2.55, *p* = .01). Lastly, there was no difference between the leader and follower composites in terms of rated happiness (*b* = 0.07, 95% CI = [−0.04, 0.18], *SE* = 0.06, *t*(712.00) = 1.30, *p* =.20) or warmth (*b* = 0.07, 95% CI = [−0.07, 0.21], *SE* = 0.07, *t*(712.00) = 0.98, *p* = .33). Note that the positive likability effect is relatively small compared to the other dimensions such as powerfulness (i.e., comparing *b*s and their confidence intervals).

The overall analysis revealed that leader images were rated more highly on the ability dimensions, regardless of whether the image was the base image, a random slice of the base image, or generated by female or male participants. Given the strong association of “think leader, think White; think manager, think male,” it is unsurprising that the average mental representation of a leader appears to be a White male. However, this association between leadership and masculinity has been found to be stronger among male participants than female participants [17]. To examine this masculinity association across participant gender, we conducted interaction analyses between the gender of the image-generating participants and leader/follower status, followed by simple effects analyses comparing leader and follower images generated by female and male participants separately.

These analyses revealed several significant interactions between the gender of the image-generating participants and leader/follower status across several different dimensions. For the perceived demographics measures, while both female and male participant-generated images showed leaders as more male and White than followers, there was a significant interaction for race (*b* =  −1.10, 95% CI =  [−1.90, −0.30], *SE* =  0.40, *t*(87.00) =  −2.76, *p* = .007), indicating that this White-leader association was stronger in male participant-generated images than female participant-generated images (Female: *b* =  −2.10, 95% CI =  [−2.66, − 1.54], *SE* = 0.29, *t*(58.00) =  −7.33, *p* < .001; Male: *b* =  −1.00, 95% CI =  [−1.61, −0.39], *SE* = 0.31, *t*(58.00) =  −3.2, *p* = .002). No interaction was found on the gender dimension (See Table 2).

**Table 2:** Descriptive statistics and regression results for the female and male participant-generated leader and follower ratings. Study 1c.

Panel A: Perceived Demographics						
Dimension	Generated by	Condition	Mean^b^	SD	Interaction Results	Regression Results
gender^a^	Female	leader	1.89	1.37	b = −0.39, 95% CI = [−1.22, 0.44], SE = 0.42, t(81.00) = −0.95, p = .35	b = −3.14, 95% CI = [−3.8, −.48], SE = 0.34, t(54.00) = −9.34, p < .001
		follower	5.04	1.14		
	Male	leader	1.43	0.88		b = −3.54, 95% CI = [−4.05, − 3.02], SE = 0.26, t(54.00) = −13.51, p < .001
		follower	4.96	1.07		
race	Female	leader	2.53	1.20	b = −1.10, 95% CI = [−1.90, −0.30], SE = 0.40, t(87.00) = −2.76, p = .007	b = −1.00, 95% CI = [−1.61, −0.39], SE = 0.31, t(58.00) = −3.2, p = .002
		follower	3.53	1.22		
	Male	leader	2.10	1.12		b = −2.10, 95% CI = [−0.66, − 1.54], SE = 0.29, t(58.00) = −7.33, p < .001
		follower	4.20	1.10		
**Panel B: Perceived Dominance**						
**Dimension**	**Generated by**	**Condition**	**Mean** ^b^	**SD**	**Interaction Results**	**Regression Results**
competence^c^	Female	leader	4.47	1.01	b = 0.40, 95% CI = [−0.26, 1.06], SE = 0.33, t(87.00) = 1.21, p = .23	b = 1.57, 95% CI = [1.05, 2.09], SE = 0.27, t(29.00) = 5.90, p < .001
		follower	2.90	1.18		
	Male	leader	4.60	1.04		b = 1.97, 95% CI = [1.45, 2.49], SE = 0.26, t(29.00) = 7.43, p < .001
		follower	2.63	1.10		
dominance	Female	leader	3.83	1.02	b = −0.13, 95% CI = [−0.82, 0.55], SE = 0.35, t(87.00) = −0.39, p = .70	b = 1.50, 95% CI = [1.03, 1.97], SE = 0.24, t(29.00) = 6.29, p < .001
		follower	2.33	0.96		
	Male	leader	4.23	0.86		b = 1.37, 95% CI = [0.86, 1.88], SE = 0.26, t(58.00) = 5.26, p < .001
		follower	2.87	1.14		
powerfulness	Female	leader	4.17	1.14	b = −0.17, 95% CI = [−0.95, 0.60], SE = 0.39, t(84.00) = −0.45, p = .66	b = 2.00, 95% CI = [1.43, 2.57], SE = 0.29, t(56.00) = 6.90, p < .001
		follower	2.17	1.07		
	Male	leader	4.34	0.90		b = 1.83, 95% CI = [1.30, 2.36], SE = 0.27, t(56.00) = 6.74, p < .001
		follower	2.52	1.15		
**Panel C: Perceived Positivity**						
**Dimension**	**Generated by**	**Condition**	**Mean** ^b^	**SD**	**Interaction Results**	**Regression Results**
attractiveness	Female	leader	4.34	1.11	b = 0.55, 95% CI = [−0.07, 1.18], SE = 0.31, t(84.00) = 1.76, p = .08	b = 1.41, 95% CI = [0.96, 1.86], SE = 0.23, t(28.00) = 6.14, p < .001
		follower	2.93	1.41		
	Male	leader	4.34	0.97		b = 1.97, 95% CI = [1.44, 2.49], SE = 0.27, t(28.00) = 7.29, p < .001
		follower	2.38	1.50		
happiness	Female	leader	4.31	0.76	b = 0.72, 95% CI = [0.16, 1.29], SE = 0.28, t(84.00) = 2.57, p = .01	b = −0.17, 95% CI = [−0.57, 0.23], SE = 0.21, t(28.00) = −0.84, p = .41
		follower	4.48	0.83		
	Male	leader	3.24	0.69		b = 0.55, 95% CI = [0.16, 0.95], SE = 0.20, t(28.00) = 2.73, p = .01
		follower	2.69	1.00		
likability	Female	leader	4.52	1.06	b = 0.93, 95% CI = [0.26, 1.60], SE = 0.34, t(84.00) = 2.77, p = .007	b = 0.21, 95% CI = [−0.30, 0.72], SE = 0.26, t(28.00) = 0.78, p = .43
		follower	4.31	1.20		
	Male	leader	4.03	1.09		b = 1.14, 95% CI = [0.67, 1.60], SE = 0.24, t(28.00) = 4.81, p < .001
		follower	2.90	1.18		
trustworthiness	Female	leader	4.07	1.28	b = 1.00, 95% CI = [0.19, 1.81], SE = 0.40, t(87.00) = 2.48, p = .02	b = 0.00, 95% CI = [−0.59, 0.59], SE = 0.03, t(58.00) = 0.00, p > .99
		follower	4.07	1.05		
	Male	leader	4.00	1.08		b = 1.00, 95% CI = [0.45, 1.55], SE = 0.28, t(58.00) = 3.58, p < .001
		follower	3.00	1.08		
warmth	Female	leader	4.00	0.85	b = 0.93, 95% CI = [0.22, 1.64], SE = 0.36, t(84.00) = 2.61, p = .01	b = −0.28, 95% CI = [−0.72, 0.17], SE = 0.24, t(28.00) = −1.22, p = .23
		follower	4.28	1.28		
	Male	leader	3.14	1.09		b = 0.66, 95% CI = [0.19, 1.12], SE = 0.24, t(28.00) = 2.74, p = .01
		follower	2.48	1.09		

^a^Higher ratings in Gender indicate the image was more likely perceived as Female, and higher ratings in Race indicate the image was more likely perceived as African American.

^b^All ratings are on a 1–6 point scale.

For example, 1 means “Definitely MALE/ WHITE AMERICAN” and 6 means “Definitely FEMALE/ AFRICAN AMERICAN” in the perceived demographics ratings.

^c^In the other ratings, 1 means “Definitely INCOMPETENT/SUBMISSIVE/ POWERLESS/ UNATTRACTIVE/ UNHAPPY/ UNLIKABLE/ UNTRUSTWORTHY/ COLD” and 6 means “Definitely COMPETENT/ DOMINANT/ PWERFUL/ ATTRACTIVE/ HAPPY/ LIKABLE/ TRUSTWORTHY/ WARM”.

For the items on the dominance dimension, the results showed no interaction effect, suggesting that both female and male participants associated leadership with competence, dominance, and powerfulness (See Table 2). However, for several of the positivity dimension items, the results showed significant interaction effects. For instance, the happiness ratings showed a significant interaction (*b* =  0.72, 95% CI = [0.16, 1.29], *SE* =  0.28, *t*(84.00) =  2.57, *p* = .01) with female participant-generated leader-follower images not differing significantly in happiness (*b* =  −0.17, 95% CI = [−0.57, 0.23], *SE* =  0.21, *t*(28.00) =  −0.84, *p* = .41), but the male-generated leader image received a more positive happiness rating than the follower image (*b* =  0.55, 95% CI = [0.16, 0.95], *SE* =  0.20, *t*(28.00) =  2.73, *p* = .01).

Similar patterns emerged for likability (interaction: *b* =  0.93, 95% CI = [0.26, 1.60], *SE* =  0.34, *t*(84.00) =  2.77, *p* = .007), trustworthiness (interaction: *b* =  1.00, 95% CI = [0.19, 1.81], *SE* =  0.40, *t*(87.00) =  2.48, *p* = .02), and warmth (interaction: *b* =  0.93, 95% CI = [0.22, 1.64], *SE* =  0.36, *t*(84.00) =  2.61, *p* = .01). Across all three dimensions, female participants generated leader-follower images that didn’t differ significantly (likability: *b* =  0.21, 95% CI = [−0.30, 0.72], *SE* =  0.26, *t*(28.00) =  0.78, *p* = .43; trustworthiness: *b* =  0.00, 95% CI = [−0.59, 0.59], *SE* =  0.03, *t*(58.00) =  0, *p* > .99; warmth: *b* =  − 0.28, 95% CI = [−0.72, 0.17], *SE* =  0.24, *t*(28.00) =  − 1.22, *p* = .23) while male-genera*t*ed leader images were consistently rated more positively than follower images (likability: *b* =  1.14, 95% CI = [0.67, 1.60], *SE* =  0.24, *t*(28.00) =  4.81, *p* < .001; trustworthiness: *b* =  1.00, 95% CI = [0.45, 1.55], *SE* =  0.28, *t*(58.00) =  3.58, *p* < .001; warmth: *b* =  0.66, 95% CI = [0.19, 1.12], *SE* =  0.24, *t*(28.00) =  2.74, *p* = .01).

When visualizing a leader’s face that has a demographically ambiguous slate (i.e., no clear gender or race), compared to visualizing a follower, the resulting average image (which captures the average perceptual representation) looks more White and male and conveys competence, dominance, and power, and overall displays no more strong positivity features besides—to a small degree—attractiveness and likability. This trend intensifies in the male participant-generated images. In the eyes of male participants, the imagined leader’s face was significantly happier, more likable, trustworthy and warm compared to follower images, while female participants did not imagine leaders as showing these positivity dimensions more than followers.

## Study 2: what does a male leader look like?

In Study 2, we sought to understand the role of gender in perceptual imprinting by using a *race-ambiguous but clearly male* base image, from which we generated composite images of leaders and followers. Thus, Study 2 allowed us to infer the perceptual imprint of male leaders, specifically.

### Methods

**Fig 2:**
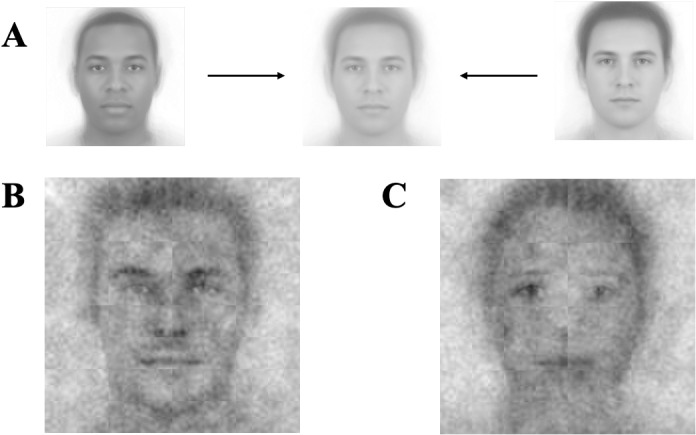
Visual composites for Study 2. Image attribution: “Ma, Correll, & Wittenbrink (2015). The Chicago Face Database: A Free Stimulus Set of Faces and Norming Data. Behavior Research Methods, 47, 1122-1135.” Reprinted from Chicago Face Database under a CC BY license, with permission from The University of Chicago Center for Decision Science, original copyright 2015. (A) Male Composite Image. (B) Visual composite of all leader selections. (C) Visual composite of all follower selections.

The procedures of Studies 2a–2c were identical to Studies 1a–1c, with the only difference being that the base image (with visual noise superimposed upon) was clearly male, but race-ambiguous. Two hundred and forty-seven participants completed Study 2a, where participants selected, across 300 trials, which of two images looked more like a leader (*M* =  39.49 years, *SD* =  13.56 years, 124 women, 119 men; 73.25% White, 13.17% Black, 5.35% Hispanic, 4.53% Asian, and 3.7% other racial background participants), and 251 participants completed Study 2b, selecting across the 300 trials which image looks more like a follower (*M* =  40.94 years, *SD* =  13.83 years, 119 women, 127 men; 75.4% White, 8.06% Black, 6.85% Hispanic, 7.26% Asian, and 2.42% other racial background participants). To ensure the morphed image—used for the base image—was truly race-ambiguous, we pretested several versions of the morphed image and selected the one that participants perceived as most ambiguous in terms of race (see Fig 2 for the morphed image and SOM for the pretesting procedures). Four participants were excluded in Study 2a, and three participants were excluded in Study 2b, due to the same exclusion criteria as in Studies 1a and 1b.

Similar to Study 1c, in Study 2c, we sought 30 participants per each rating (meeting the needed 20–40 ratings per stimulus for reliable means; [31]). Again, each participant rated the 26 composite images on one randomly selected dimension of the ten (300 participants completed the survey and 3 were excluded, *M* =  38.80 years, *SD* =  12.09 years, 151 women, 145 men and 1 other; 67.68% White, 12.79% Black, 7.74% Hispanic, 10.44% Asian, and 1.35% other racial background participants), thus yielding about 30 participants per each rating. Five participants were excluded due to the same exclusion criteria as in Study 1c.

### Results and discussion

As in Study 1, we again implemented multilevel models, including random intercepts for image and rater. The new leader and follower composite images generated from participants in Study 2a and Study 2b are presented in Fig 2 and were rated by participants in Study 2c.

Consistent with Study 1, but now constrained to male targets, the leader composite images appeared to be more White than the follower composite images (*b* =  −1.56, 95% CI =  [−1.70, − 1.42], *SE* =  0.07, *t*(762.00) =  −21.70, *p* <  0.001). Although the base images were male images, there was a difference in perceived gender between the leader and follower images as well. The leader composite images were rated to be more male than the follower composite images (*b* =  −2.93, 95% CI =  [−3.08, − 2.77], *SE* =  0.06, *t*(737.00) =  −37.09, *p* <  0.001). The descriptive statistics of these measures can be found in Table 3.

**Table 3:** Descriptive statistics for the leader and follower ratings. Study 2c.

Category	Dimension	Condition	Mean^b^	SD
Perceived Demographics	gender^a^	leader	1.40	0.64
		follower	4.59	0.99
	race	leader	2.46	1.30
		follower	4.02	0.99
Perceived Dominance	competence^c^	leader	4.24	1.12
		follower	3.32	1.25
	dominance	leader	4.56	1.05
		follower	2.36	1.08
	powerfulness	leader	4.44	0.82
		follower	2.60	1.03
Perceived Positivity	attractiveness	leader	4.22	1.11
		follower	2.56	1.08
	happiness	leader	3.85	0.83
		follower	3.09	0.91
	likability	leader	4.14	1.22
		follower	3.60	1.26
	trustworthiness	leader	3.45	1.20
		follower	3.17	1.28
	warmth	leader	3.66	1.29
		follower	3.33	1.17

^a^Higher ratings in Gender indicate the image was more likely perceived as Female, and higher ratings in Race indicate the image was more likely perceived as African American.

^b^All ratings are on a 1–6 point scale.

For example, 1 means “Definitely MALE/ WHITE AMERICAN” and 6 means “Definitely FEMALE/ AFRICAN AMERICAN” in the perceived demographics ratings.

^c^In the other ratings, 1 means “Definitely INCOMPETENT/SUBMISSIVE/ POWERLESS/ UNATTRACTIVE/ UNHAPPY/ UNLIKABLE/ UNTRUSTWORTHY/ COLD” and 6 means “Definitely COMPETENT/ DOMINANT/ PWERFUL/ ATTRACTIVE/ HAPPY/ LIKABLE/ TRUSTWORTHY/ WARM”.

Aligned with Study 1 (but using composites generated from male targets), relative to follower composites, the leader composites received more favorable ratings for items that represent dominance: more competent (*b* =  0.92, 95% CI =  [0.79, 1.05], *SE* =  0.07, *t*(737.00) =  14.03, *p* <  0.001), more dominant (*b* =  2.19, 95% CI =  [2.06, 2.33], *SE* =  0.07, *t*(662.00) =  32.69, *p* <  0.001), and more powerful (*b* =  1.83, 95% CI =  [1.71, 1.95], *SE* =  0.06, *t*(737.00) =  29.87, *p* <  0.001).

Relative to the male follower composites, the male leader composites received more favorable ratings for items that represent positivity: more attractive (*b* =  1.66, 95% CI =  [1.53, 1.78], *SE* =  0.06, *t*(762.00) =  26.25, *p* <  0.001), more happy (*b* =  0.77, 95% CI =  [0.67, 0.87], *SE* =  0.05, *t*(737.00) =  14.79, *p* <  0.001), more likable (*b* =  0.54, 95% CI =  [0.41, 0.67], *SE* =  0.07, *t*(737.00) =  8.00, *p* <  0.001), more trustworthy (*b* =  0.28, 95% CI =  [0.15, 0.42], *SE* =  0.07, *t*(712.00) =  4.05, *p* <  0.001), and more warm (*b* =  0.34, 95% CI =  [0.18, 0.49], *SE* =  0.08, *t*(687.00) =  4.33, *p* <  0.001). Note that the favorable ratings on the positivity dimensions (except for attractiveness) are relatively small compared to the other dimensions (i.e., comparing *b*s and their confidence intervals).

The current study utilized a male composite base image, aligning the social identity of the base image with the male participants’ mental representations. To examine the differences between female and male participants’ mental representations of male leaders, we conducted interaction analyses between the image-generating participant gender and leader/follower status, followed by simple effects analyses. While there were no interaction effects on the demographics and competence dimensions, there were significant interaction effects on the positivity dimensions (See Table 4).

**Table 4:** Descriptive statistics and regression results for the female and male participant-generated leader and follower ratings. Study 2c.

Panel A: Perceived Demographics						
Dimension	Generated by	Condition	Mean^b^	SD	Interaction Results	Regression Results
gender^a^	Female	leader	1.23	0.50	b = −0.03, 95% CI = [−0.48, 0.42], SE = 0.23, t(90.00) = −0.14, p = .89	b = −3.65, 95% CI = [−3.98, −3.31], SE = 0.17, t(30.00) = 0.17, p < .001
		follower	4.87	0.88		
	Male	leader	1.29	0.53		b = −3.68, 95% CI = [−3.99, −3.37], SE = 0.16, t(60.00) = 0.16, p < .001
		follower	4.97	0.71		
race	Female	leader	2.39	1.41	b = 0.00, 95% CI = [−0.72, 0.72], SE = 0.37, t(90.00) = 0.00, p >.99	b = −0.61, 95% CI = [−0.19, − 1.03], SE = 0.30, t(30.00) = 0.30, p < .001
		follower	4.00	0.97		
	Male	leader	2.35	1.14		b = −1.61, 95% CI = [−2.16, −1.07], SE = 0.28, t(60.00) = 0.28, p < .001
		follower	3.97	1.05		
**Panel B: Perceived Dominance**						
**Dimension**	**Generated by**	**Condition**	**Mean** ^b^	**SD**	**Interaction Results**	**Regression Results**
competence^c^	Female	leader	4.43	1.14	b = 0.33, 95% CI = [−0.33, 0.99], SE = 0.34, t(87.00) = 0.98, p = .33	b = 1.00, 95% CI = [0.48, 1.52], SE = 0.27, t(29.00) = 0.27, p < .001
		follower	3.43	1.19		
	Male	leader	4.37	1.07		b = 1.33, 95% CI = [0.81, 1.86], SE = 0.27, t(29.00) = 0.27, p < .001
		follower	3.03	1.33		
dominance	Female	leader	4.52	0.98	b = 0.26, 95% CI = [−0.35, 0.87], SE = 0.31, t(78.00) = 0.84, p = .40	b = 2.37, 95% CI = [1.92, 2.82], SE = 0.23, t(26.00) = 0.23, p < .001
		follower	2.15	0.91		
	Male	leader	4.70	0.91		b = 2.63, 95% CI = [2.18, 3.08], SE = 0.23, t(52.00) = 0.23, p < .001
		follower	2.07	0.78		
powerfulness	Female	leader	4.60	0.86	b = 0.37, 95% CI = [−0.26, 1.00], SE = 0.32, t(87.00) = 1.14, p = .26	b = 1.70, 95% CI = [1.18, 2.22], SE = 0.27, t(29.00) = 0.27, p < .001
		follower	2.90	1.24		
	Male	leader	4.43	0.73		b = 2.07, 95% CI = [1.67, 2.47], SE = 0.20, t(58.00) = 10.11, p < .001
		follower	2.37	0.85		
**Panel C: Perceived Positivity**						
**Dimension**	**Generated by**	**Condition**	**Mean** ^b^	**SD**	**Interaction Results**	**Regression Results**
attractiveness	Female	leader	4.61	0.99	b = −0.19, 95% CI = [−0.83, 0.44], SE = 0.33, t(90.00) = −0.60, p = .55	b = 1.94, 95% CI = [1.44, 2.43], SE = 0.25, t(30.00) = 0.25, p < .001
		follower	2.68	1.05		
	Male	leader	4.29	0.86		b = 1.74, 95% CI = [1.24, 2.24], SE = 0.25, t(60.00) = 0.25, p < .001
		follower	2.55	1.12		
happiness	Female	leader	4.30	0.84	b = 0.97, 95% CI = [0.47, 1.47], SE = 0.25, t(87.00) = 3.85, p < .001	b = 0.33, 95% CI = [−0.01, 0.68], SE = 0.18, t(29.00) = 1.90, p = .07
		follower	3.97	0.76		
	Male	leader	3.83	0.65		b = 1.30, 95% CI = [0.92, 1.68], SE = 0.19, t(29.00) = 6.75, p < .001
		follower	2.53	0.97		
likability	Female	leader	4.37	1.43	b = 0.37, 95% CI = [−0.35, 1.09], SE = 0.37, t(87.00) = 1.00, p = .32	b = 0.37, 95% CI = [−0.24, 0.97], SE = 0.31, t(29.00) = 1.19, p = .25
		follower	4.00	1.20		
	Male	leader	4.40	1.04		b = 0.73, 95% CI = [0.32, 1.14], SE = 0.21, t(29.00) = 3.52, p = .001
		follower	3.67	1.21		
trustworthiness	Female	leader	3.69	1.26	b = 0.24, 95% CI = [−0.46, 0.94], SE = 0.36, t(84.00) = 0.67, p = .50	b = 0.24, 95% CI = [−0.30, 0.78], SE = 0.27, t(28.00) = 0.98, p = .39
		follower	3.45	1.45		
	Male	leader	3.69	1.20		b = 0.48, 95% CI = [−0.02, 0.98], SE = 0.26, t(28.00) = 1.89, p = .07
		follower	3.21	1.01		
warmth	Female	leader	4.04	1.10	b = 1.07, 95% CI = [0.32, 1.82], SE = 0.38, t(81.00) = 2.79, p = .01	b = −0.25, 95% CI = [−0.79, 0.29], SE = 0.27, t(54.00) = −0.91, p = .37
		follower	4.29	0.94		
	Male	leader	3.86	1.21		b = 0.82, 95% CI = [0.19, 1.45], SE = 0.32, t(27.00) = 2.56, p < .02
		follower	3.04	1.26		

^a^Higher ratings in Gender indicate the image was more likely perceived as Female, and higher ratings in Race indicate the image was more likely perceived as African American.

^b^All ratings are on a 1–6 point scale.

For example, 1 means “Definitely MALE/ WHITE AMERICAN” and 6 means “Definitely FEMALE/ AFRICAN AMERICAN” in the perceived demographics ratings.

^c^In the other ratings, 1 means “Definitely INCOMPETENT/SUBMISSIVE/ POWERLESS/ UNATTRACTIVE/ UNHAPPY/ UNLIKABLE/ UNTRUSTWORTHY/ COLD” and 6 means “Definitely COMPETENT/ DOMINANT/ PWERFUL/ ATTRACTIVE/ HAPPY/ LIKABLE/ TRUSTWORTHY/ WARM”.

For happiness and warmth ratings, there were significant interactions between participant gender and leader/follower status (happiness: *b* =  0.97, 95% CI [0.47, 1.47], *SE* =  0.25, *t*(87.00) =  3.85, *p* < .001; warmth: *b* =  1.07, 95% CI [0.32, 1.82], *SE* =  0.38, *t*(81.00) =  2.79, *p* = .01). The patterns of the interaction effects were similar on these two dimensions such that the female participant-generated composite images showed a smaller or no difference between leaders and followers (happiness: *b* =  0.33, 95% CI [−0.01, 0.68], *SE* =  0.18, *t*(29.00) =  1.90, *p* = .07; warmth: *b* =  − 0.25, 95% CI [−0.79, 0.29], *SE* =  0.27, *t*(54.00) =  − 0.91, *p* = .37), but the male participant-generated composite images showed significant positive differences between leaders and followers on these dimensions (happiness: *b* =  1.30, 95% CI [0.92, 1.68], *SE* =  0.19, *t*(29.00) =  6.75, *p* < .001; warmth: *b* =  0.82, 95% CI [0.19, 1.45], *SE* =  0.32, *t*(27.00) =  2.56, *p* = .02).

Thus, when choosing between two randomly generated *male* images, relative to followers, people imagined leaders to look more White, male, and dominant/ competent/ powerful (i.e., able), and to a lesser degree, positive. However, it is worth noting that some of the positivity effects were not observed in the female participant-generated composite images.

In Studies 1 and 2, compared to imagined followers, leaders appeared to be a White male, and male participants also expected the leader to appear both dominant/competent/powerful (i.e., able) and positive, whereas female participants only expected the leader to able.

## Study 3: What does a female leader look like?

Studies 1 and 2 produced similar results, especially on the demographic and the ability measures, despite the composite images in Study 1 being generated from a gender- and race-ambiguous base image, whereas Study 2 used a clearly male base image. These findings suggest that compared to followers, the *perceptual imprint* of a leader is a White male who looks clearly dominant.

What does this mean for women? Prior work exploring judgments of known others finds that women need to be perceived as both able and positive to be judged as effective leaders [17]. This suggests that when we constrain imagined targets to be female, different features would come to mind than those observed in Studies 1 and 2.

### Methods

The procedures of Studies 3a–3c were identical to Studies 2a–2c, with the only difference being that the base image (with visual noise superimposed upon) was clearly female, and again, race ambiguous. Two hundred and fifty-two participants completed Study 3a, where participants selected, across 300 trials, which of two images looked more like a leader (*M* =  39.36 years, *SD* =  12.75 years, 125 women, 127 men;71.03% White, 12.7% Black, 5.95% Hispanic, 5.56% Asian, and 4.76% other racial background participants), and 233 participants completed Study 3b, selecting across the 300 trials which image looks more like a follower (*M* =  40.45 years, *SD* =  12.99 years, 113 women, 118 men, and 2 non-binary; 72.53% White, 9.87% Black, 8.15% Hispanic, 6.44% Asian, and 3% other racial background participants). Four participants were excluded in Study 3b, due to the same exclusion criteria as in Studies 1a and 1b.

**Fig 3:**
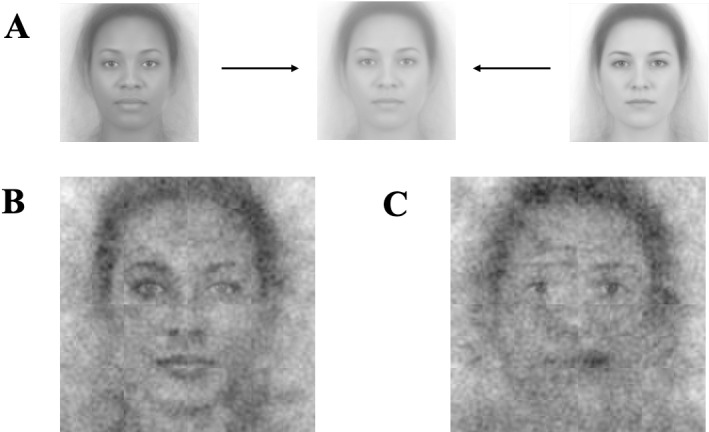
Female visual composites for Study 3. Image attribution: “Ma, Correll, & Wittenbrink (2015). The Chicago Face Database: A Free Stimulus Set of Faces and Norming Data. Behavior Research Methods, 47, 1122-1135.” Reprinted from Chicago Face Database under a CC BY license, with permission from The University of Chicago Center for Decision Science, original copyright 2015. (A) Female Composite Image. (B) Visual composite of all leader selections. (C) Visual composite of all follower selections.

In Studies 3a & 3b, the base image was derived from morphing Black female and White female images. To ensure the morphed image was truly race-ambiguous, we pretested several versions of the morphed image and selected the one that participants perceived as most ambiguous with respect to race (see Fig 3 for the morphed image and SOM for the pretesting procedures).

In Study 3c, again, each participant rated the 26 composite images on one randomly selected dimension of the ten (309 participants, *M* =  39.23 years, *SD* =  12.93 years, 157 women, 150 men and 2 other; 66.67% White, 13.47% Black, 6.73% Hispanic, 9.76% Asian, and 3.37% other racial background participants), thus yielding about 30 participants per each rating. Six participants were excluded due to the same exclusion criteria as in Study 1c.

### Results and discussion

The new leader and follower composite images generated from participants in Studies 3a and 3b are presented in Fig 3 and were rated by participants in Study 3c. As in the prior studies, we again implemented multilevel models that included random intercepts for image and rater.

With a female base image, the leader composite images were judged as appearing more White than the follower composite images, *b* =  −0.70, 95% CI =  [−0.87, − 0.53], *SE* =  0.09, *t*(712.00) =  −8.21, *p* <  0.001 and appearing more female than the follower composite images, *b* =  1.76, 95% CI =  [1.65, 1.88], *SE* =  0.06, *t*(712.00) =  29.24, *p* <  0.001. The descriptive statistics of these measures can be found in Table 5.

**Table 5:** Descriptive statistics for the leader and follower ratings. Study 3c.

Category	Dimension	Condition	Mean^b^	SD
Perceived Demographics	gender^a^	leader	5.32	0.81
		follower	3.55	1.17
	race	leader	3.16	1.30
		follower	3.86	1.18
Perceived Dominance	competence^c^	leader	4.63	1.22
		follower	3.34	1.27
	dominance	leader	3.89	1.07
		follower	2.83	1.18
	powerfulness	leader	4.05	1.03
		follower	2.42	1.03
Perceived Positivity	attractiveness	leader	4.55	1.11
		follower	2.86	0.98
	happiness	leader	3.66	0.92
		follower	3.03	1.08
	likability	leader	4.32	1.19
		follower	3.28	1.12
	trustworthiness	leader	3.97	1.16
		follower	3.33	1.24
	warmth	leader	3.48	1.31
		follower	3.15	1.29

^a^Higher ratings in Gender indicate the image was more likely perceived as Female, and higher ratings in Race indicate the image was more likely perceived as African American.

^b^All ratings are on a 1 − 6 point scale.

For example, 1 means “Definitely MALE/ WHITE AMERICAN” and 6 means “Definitely FEMALE/ AFRICAN AMERICAN” in the perceived demographics ratings.

^c^In the other ratings, 1 means “Definitely INCOMPETENT/SUBMISSIVE/ POWERLESS/ UNATTRACTIVE/ UNHAPPY/ UNLIKABLE/ UNTRUSTWORTHY/ COLD” and 6 means “Definitely COMPETENT/ DOMINANT/ PWERFUL/ ATTRACTIVE/ HAPPY/ LIKABLE/ TRUSTWORTHY/ WARM”.

Moving to the trait ratings, relative to the female follower composite images, the female leader composite images received more favorable ratings for items that represent dominance: more competent (*b* =  1.29, 95% CI =  [1.17, 1.40], *SE* =  0.06, *t*(737.00) =  21.54, *p* <  0.001), more dominant (*b* =  1.07, 95% CI =  [0.92, 1.22], *SE* =  0.08, *t*(724.00) =  13.98, *p* <  0.001), and more powerful (*b* =  1.62, 95% CI =  [1.50, 1.75], *SE* =  0.07, *t*(712.00) =  24.75, *p* <  0.001).

Relative to the female follower composite images, the female leader composite images received more favorable ratings for items that represent positivity: more attractive (*b* =  1.69, 95% CI =  [1.57, 1.81], *SE* =  0.06, *t*(737.00) =  27.25, *p* <  0.001), more likable (*b* =  0.63, 95% CI =  [0.52, 0.74], *SE* =  0.07, *t*(712.00) =  15.17, *p* <  0.001), more trustworthy (*b* =  0.64, 95% CI =  [0.52, 0.74], *SE* =  0.07, *t*(762.00) =  8.75, *p* <  0.001), and more warm (*b* =  0.33, 95% CI =  [0.17, 0.49], *SE* =  0.08, *t*(712.00) =  3.98, *p* <  0.001), but no difference on perceived happiness (*b* =  0.07, 95% CI =  [−0.04, 0.18], *SE* =  0.06, *t*(712.00) =  1.30, *p* = .20). Similar to the results from Study 2c, the favorable ratings on the positivity dimensions (except for attractiveness) are again relatively small compared to the other ability dimensions (i.e., comparing *b*s and their confidence intervals).

In the earlier two studies, the female-participant vs. male-participant generated images were not rated differently on the dominance dimensions. However, in the current study, the base image was clearly female. How do participant gender differences in the mental representation of a female leader vary across participant gender? The analysis revealed that interaction effects were found in all three categories of dependent measures (see Table 6). On the demographics dimensions, there was a significant interaction for race ratings (*b* =  −1.72, 95% CI = [−2.54, − 0.90], *SE* =  0.42, *t*(112.00) =  −4.14, *p* < .001). Simple effect analysis revealed that there was a significant leader-follower difference for male participant-generated images such that the leader was perceived as more likely to be White American than the follower (*b* =  −1.76, 95% CI [−2.33, −1.19], *SE* =  0.29, *t*(56.00) =  −6.04, *p* < .001), but no difference was found for female participant-generated image pairs (*b* =  −0.03, 95% CI [−0.62, 0.55], *SE* =  0.30, *t*(56.00) =  −0.12, *p* = .91).

**Table 6:** Descriptive statistics and regression results for the female and male participant-generated leader and follower ratings. Study 3c.

Panel A: Perceived Demographics						
Dimension	Generated by	Condition	Mean^b^	SD	Interaction Results	Regression Results
gender^a^	Female	leader	5.62	0.56	b = −0.07, 95% CI = [−0.61, 0.47], SE = 0.27, t(84.00) = −0.25, p = .80	b = 2.14, 95% CI = [1.78, 2.50], SE = 0.18, t(28.00) = 11.63, p < .001
		follower	3.48	0.99		
	Male	leader	5.45	0.69		b = 2.07, 95% CI = [1.65, 2.49], SE = 0.22, t(28.00) = 9.58, p < .001
		follower	3.38	1.12		
race	Female	leader	3.55	1.09	b = −1.72, 95% CI = [−2.54, −0.90], SE = 0.42, t(112.00) = −4.14, p < .001	b = −0.03, 95% CI = [−0.62, 0.55], SE = 0.30, t(56.00) = −0.12, p = .91
		follower	3.59	1.18		
	Male	leader	2.28	1.22		b = −1.76, 95% CI = [−2.33, −1.19], SE = 0.29, t(56.00) = −6.04, p < .001
		follower	4.03	0.98		
**Panel B: Perceived Dominance**						
**Dimension**	**Generated by**	**Condition**	**Mean** ^b^	**SD**	**Interaction Results**	**Regression Results**
competence^c^	Female	leader	4.57	1.14	b = 0.30, 95% CI = [−0.32, 0.92], SE = 0.32, t(87.00) = 0.95, p = .34	b = 1.20, 95% CI = [0.75, 1.65], SE = 0.23, t(29.00) = 5.17, p < .001
		follower	3.37	1.25		
	Male	leader	4.83	1.18		b = 1.50, 95% CI = [0.98, 2.02], SE = 0.27, t(29.00) = 5.64, p < .001
		follower	3.33	1.30		
dominance	Female	leader	3.59	1.30	b = 0.93, 95% CI = [0.12, 1.74], SE = 0.41, t(112.00) = 2.26, p = .03	b = 0.86, 95% CI = [0.27, 1.46], SE = 0.30, t(56.00) = 2.84, p < .001
		follower	2.72	1.00		
	Male	leader	4.31	1.04		b = 1.79, 95% CI = [1.25, 2.34], SE = 0.28, t(56.00) = 6.42, p < .001
		follower	2.52	1.09		
powerfulness	Female	leader	4.07	1.22	b = 0.45, 95% CI = [−0.29, 1.19], SE = 0.38, t(84.00) = 1.20, p = .24	b = 1.55, 95% CI = [1.10, 2.00], SE = 0.23, t(28.00) = 6.73, p < .001
		follower	2.52	0.83		
	Male	leader	4.21	1.15		b = 2.00, 95% CI = [1.44, 2.56], SE = 0.28, t(56.00) = 7.04, p < .001
		follower	2.21	1.01		
**Panel C: Perceived Positivity**						
**Dimension**	**Generated by**	**Condition**	**Mean** ^b^	**SD**	**Interaction Results**	**Regression Results**
attractiveness	Female	leader	4.50	1.17	b = 0.87, 95% CI = [0.25, 1.49], SE = 0.31, t(87.00) = 2.77, p = .01	b = 1.47, 95% CI = [1.04, 1.89], SE = 0.22, t(29.00) = 6.72, p < .001
		follower	3.03	0.67		
	Male	leader	4.80	0.92		b = 2.33, 95% CI = [1.88, 2.79], SE = 0.23, t(58.00) = 10.12, p < .001
		follower	2.47	0.86		
happiness	Female	leader	4.58	0.89	b = −0.03, 95% CI = [−1.65, − 0.41], SE = 0.32, t(90.00) = −0.25, p = .002	b = 1.16, 95% CI = [0.75, 1.57], SE = 0.21, t(30.00) = 5.59, p < .001
		follower	3.42	1.18		
	Male	leader	2.77	0.99		b = 0.13, 95% CI = [−0.34, 0.60], SE = 0.24, t(30.00) = 0.54, p = .60
		follower	2.65	1.08		
likability	Female	leader	4.70	0.99	b = −0.33, 95% CI = [−1.04, 0.38], SE = 0.36, t(87.00) = − 0.92, p = .36	b = 1.50, 95% CI = [1.06, 1.94], SE = 0.22, t(29.00) = 6.71, p < .001
		follower	3.20	1.10		
	Male	leader	4.10	1.42		b = 1.17, 95% CI = [0.63, 1.71], SE = 0.28, t(29.00) = 4.23, p < .001
		follower	2.93	0.91		
trustworthiness	Female	leader	3.77	1.48	b = 0.19, 95% CI = [−0.65, 1.03], SE = 0.43, t(90.00) = 0.45, p = .66	b = 0.39, 95% CI = [−0.32, 1.10], SE = 0.36, t(30.00) = 1.07, p = 0.29
		follower	3.39	1.48		
	Male	leader	3.58	1.15		b = 0.58, 95% CI = [−0.01, 1.17], SE = 0.3, t(60.00) = 1.94, p = 0.06
		follower	3.00	1.21		
warmth	Female	leader	3.79	1.42	b = −0.28, 95% CI = [−1.21, 0.65], SE = 0.48, t(84.00) = −0.58, p = .56	b = 0.28, 95% CI = [−0.37, 0.92], SE = 0.33, t(28.00) = 0.84, p = 0.41
		follower	3.52	1.38		
	Male	leader	2.83	1.42		b = 0.00, 95% CI = [−0.67, 0.67], SE = 0.34, t(56.00) = 0.00, p > .99
		follower	2.83	1.17		

^a^Higher ratings in Gender indicate the image was more likely perceived as Female, and higher ratings in Race indicate the image was more likely perceived as African American.

^b^All ratings are on a 1 − 6 point scale.

For example, 1 means “Definitely MALE/ WHITE AMERICAN” and 6 means “Definitely FEMALE/ AFRICAN AMERICAN” in the perceived demographics ratings.

^c^In the other ratings, 1 means “Definitely INCOMPETENT/SUBMISSIVE/ POWERLESS/ UNATTRACTIVE/ UNHAPPY/ UNLIKABLE/ UNTRUSTWORTHY/ COLD” and 6 means “Definitely COMPETENT/ DOMINANT/ PWERFUL/ ATTRACTIVE/ HAPPY/ LIKABLE/ TRUSTWORTHY/ WARM”.

On the ability measures, there was a significant interaction effect on perceived dominance (*b* =  0.93, 95% CI = [0.12, 1.74], *SE* =  0.41, *t*(112.00) =  2.26, *p* = .03) such that male participants had a mental representation of female leaders being more dominant than female followers (*b* =  1.79, 95% CI = [1.25, 2.34], *SE* =  0.28, *t*(56.00) =  6.42, *p* < .001), and the effect was stronger than that of female participant-generated image pairs (*b* =  0.86, 95% CI = [0.27, 1.46], *SE* =  0.30, *t*(56.00) =  2.84, *p* < .001). On the positivity measures, significant interaction effects were found on perceived attractiveness and happiness (attractiveness: *b* =  0.87, 95% CI = [0.25, 1.49], *SE* =  0.31, *t*(87.00) =  2.77, *p* = .01; happiness: *b* =  − 1.03, 95% CI = [−1.65, − 0.41], *SE* =  0.32, *t*(90.00) =  − 3.25, *p* = .002).

Simple effects analyses showed different patterns on the two dimensions. For perceived attractiveness, both female and male participant-generated leader images received significantly higher ratings than follower images on perceived attractiveness, but the magnitude of the effect was larger for male participant-generated images than those of female participants (female: *b* =  1.47, 95% CI = [1.04, 1.89], *SE* =  0.22, *t*(29.00) =  6.72, *p* < .001; male: *b* =  2.33, 95% CI = [1.88, 2.79], *SE* =  0.23, *t*(58.00) =  10.12, *p* < .001). However, for perceived happiness, the difference between leader and follower images was significant only for female participant-generated images, where leaders were rated as happier than followers, but not for male participant-generated images (female: *b* =  1.16, 95% CI = [0.75, 1.57], *SE* =  0.21, *t*(30.00) =  5.59, *p* < .001; male: *b* =  0.13, 95% CI = [−0.34, 0.60], *SE* =  0.24, *t*(30.00) =  0.54, *p* = .60).

Study 3 was the first study thus far where there was an interaction effect on a measure from the perceived ability dimensions. This finding suggests that when it comes to the perception of female leaders, participants hold more varied mental representations of how a leader would look in terms of their ability, unlike the earlier two studies where both gender-generated images clearly were rated as high on the ability dimension.

When constraining imagined faces to be female, just as with Studies 1 and 2, we see that compared to imagined followers, the imagined leaders look more dominant, competent, and powerful. Thus, just as with imagined leaders (Study 1), and imagined male leaders (Study 2), to look leader-like women need to look dominant and able. In these studies, to a lesser degree, male participant-imagined leaders also looked more positive than imagined followers, however in the current study with imagined female leaders, the results showed a different pattern. In Study 3, both female and male participants imagined female leaders looked *more likable* than imagined female followers, and female participants also expected female leaders to look happier than female followers. This aligns with ratings of known others (i.e., whereby to perceived as effective leaders, women need to be judged as able and positive [33]. We extend this prior work to perceptual expectations. In other words, whereas male leaders need to *look* dominant and do not need to look positive in everyone’s eyes, to seem leader-like, women need to *look* dominant and likable in everyone’s eyes.

## Study 4: what does a white leader look like?

Across Studies 1-3, people believe that relative to followers, leaders’ faces look competent, dominant, and powerful, yet the composite images created by participants differed when the images were constrained to be male versus female. Male composite leader images looked no more likable and warm than male followers in female participants’ minds, whereas female composite leader images looked more likable than female followers in everyone’s minds. Aside from the small degree that men imagine male leaders to look more positive than male followers, in both men’s and women’s eyes, men largely only needed to look *able* to look like leaders, whereas women always needed to look *warm* and *able* to look like leaders.

Studies 4 and 5 took the same approach as Studies 2 and 3, but explored the influence of target race.

### Methods

The procedures of Studies 4a-4c were identical to Studies 2a-3c, with the only difference being that the base image (with visual noise superimposed upon) was now clearly White, but gender-ambiguous; 245 participants completed Study 4a, where participants selected, across 300 trials, which of two images looked more like a leader (*M* = 36.81 years, *SD* =  13.26 years, 127 women, 118 men; 71.02% White, 11.43% Black, 6.94% Hispanic, 7.35% Asian, and 3.27% other racial background participants), and 208 participants completed Study 4b, selecting across the 300 trials which image looks more like a follower (*M* =  37.34 years, *SD* =  12.79 years, 121 women, 124 men, and 1 non-binary; 70.33% White, 10.57% Black, 4.88% Hispanic, 10.57% Asian, and 3.66% other racial background participants). Four participants were excluded in Study 4a, and 3 participants were excluded in Study 4b, due to the same exclusion criteria as in Studies 1a and 1b.

**Fig 4:**
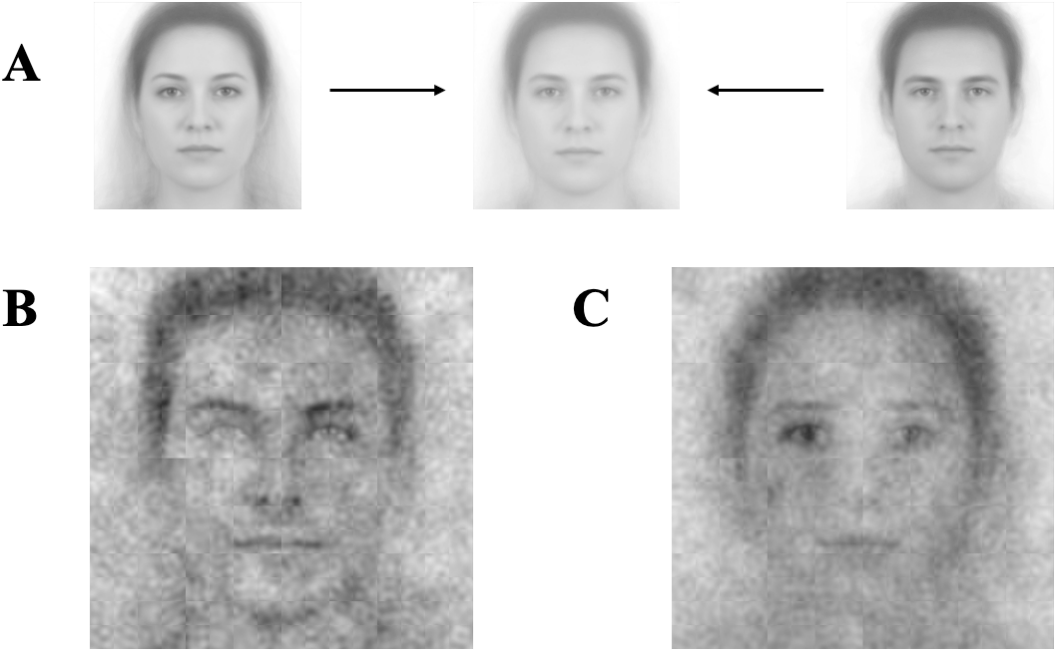
Visual composites for Study 4. Image attribution: “Ma, Correll, & Wittenbrink (2015). The Chicago Face Database: A Free Stimulus Set of Faces and Norming Data. Behavior Research Methods, 47, 1122-1135.” Reprinted from Chicago Face Database under a CC BY license, with permission from The University of Chicago Center for Decision Science, original copyright 2015. (A) White Composite Image. (B) Visual composite of all leader selections. (C) Visual composite of all follower selections.

In Studies 4a & 4b, the base image was derived from morphing White female and White male images to create the White base image. To ensure the morphed image was truly gender-ambiguous, we pretested several versions of the morphed image and selected the one that participants perceived as most ambiguous with respect to gender (see Fig 4 for the morphed image and SOM for the pretesting procedures).

In Study 4c, again, each participant rated the 26 composite images on one randomly selected dimension of the ten (301 participants, *M* =  38.49 years, *SD* =  13.03 years, 151 women, 150 men; 71.43% White, 11.96% Black, 6.31% Hispanic, 7.31% Asian, and 2.99% other racial background participants), thus yielding about 30 participants per each rating. Five participants were excluded due to the same exclusion criteria as in Study 1c.

### Results and discussion

As in the earlier studies, we implemented multilevel models, including random intercepts for image and rater.

The White leader composite was judged as appearing more male than the White follower composite, *b* = −2.64, 95% CI = [−2.79, −2.50], *SE* = 0.07, *t*(737.00) = −35.60, *p* < 0.001. Although the base image was a White image, the leader composite was judged as appearing more White than the follower composite (*b* = −0.58, 95% CI = [−0.72, −0.45], *SE* = 0.07, *t*(712.00) = −8.62, *p* < 0.001). The descrip*t*ive statistics of these measures can be found in Table 7.

**Table 7:** Descriptive statistics for the leader and follower ratings. Study 4c.

Category	Dimension	Condition	Mean^b^	SD
Perceived Demographics	gender^a^	leader	2.34	1.40
		follower	4.98	0.98
	race	leader	2.20	1.20
		follower	2.78	1.34
Perceived Dominance	competence^c^	leader	4.45	1.07
		follower	3.89	1.11
	dominance	leader	4.47	0.94
		follower	2.74	1.11
	powerfulness	leader	4.07	1.05
		follower	2.72	1.02
Perceived Positivity	attractiveness	leader	4.18	1.12
		follower	3.62	1.24
	happiness	leader	3.59	0.92
		follower	3.15	1.10
	likability	leader	3.72	0.99
		follower	3.62	1.07
	trustworthiness	leader	3.29	1.15
		follower	3.77	1.24
	warmth	leader	3.35	1.28
		follower	3.33	1.29

^a^Higher ratings in Gender indicate the image was more likely perceived as Female, and higher ratings in Race indicate the image was more likely perceived as African American.

^b^All ratings are on a 1-6 point scale.

For example, 1 means “Definitely MALE/ WHITE AMERICAN” and 6 means “Definitely FEMALE/ AFRICAN AMERICAN” in the perceived demographics ratings.

^c^In the other ratings, 1 means “Definitely INCOMPETENT/SUBMISSIVE/ POWERLESS/ UNATTRACTIVE/ UNHAPPY/ UNLIKABLE/ UNTRUSTWORTHY/ COLD” and 6 means “Definitely COMPETENT/ DOMINANT/ PWERFUL/ ATTRACTIVE/ HAPPY/ LIKABLE/ TRUSTWORTHY/ WARM”.

Relative to the White follower composites, the White leader composites received more favorable ratings for items that represent dominance: more competent (*b* =  0.56, 95% CI =  [0.44, 0.67], *SE* =  0.06, *t*(762.00) =  9.75, *p* <  0.001), more dominant (*b* =  1.74, 95% CI =  [1.60, 1.87], *SE* =  0.07, *t*(737.00) =  25.23, *p* <  0.001), and more powerful (*b* =  1.35, 95% CI =  [1.25, 1.46], *SE* =  0.06, *t*(737.00) =  24.44, *p* <  0.001).

Relative to the White follower composites, the White leader composites received less favorable rating on trustworthy (*b* =  − 0.48, 95% CI =  [−0.61, −0.36], *SE* =  0.06, *t*(762.00) =  − 7.64, *p* <  0.001), no different ratings on likability (*b* =  0.09, 95% CI =  [−0.02, 0.22], *SE* =  0.06, *t*(712.00) =  1.57, *p* = .12) or warmth (*b* =  0.02, 95% CI =  [−0.13, 0.16], *SE* =  0.07, *t*(737.00) =  0.24, *p* = .81), but more favorable ratings on attractiveness (*b* =  0.56, 95% CI =  [0.43, 0.70], *SE* =  0.07, *t*(712.00) =  8.60, *p* <  0.001), and happiness (*b* =  0.44, 95% CI =  [0.31, 0.56], *SE* =  0.06, *t*(762.00) =  7.14, *p* <  0.001). The perceived positivi*t*y items in this study show a more mixed pattern, with the favorable ratings on the positivity dimensions being again relatively small compared to the other dimensions (i.e., comparing *b*s and their confidence intervals), as in the earlier studies.

After conducting the interaction analysis to compare the female and male participant-generated images, the results showed a pattern that was similar to Studies 1 and 2. No interaction effects were found on the perceived demographics and perceived ability dimensions. However, several interaction effects were found among the positivity dimensions (See Table 8).

**Table 8:** Descriptive statistics and regression results for the female and male participant-generated leader and follower ratings. Study 4c.

Panel A: Perceived Demographics						
Dimension	Generated by	Condition	Mean^b^	SD	Interaction Results	Regression Results
gender^a^	Female	leader	2.03	1.45	b = −0.13, 95% CI = [−0.78, 0.52], SE = 0.33, t(87.00) = −0.40, p = .69	b = −0.47, 95% CI = [−0.05, 0.58], SE = 0.30, t(58.00) = −1.69, p < .001
		follower	5.50	0.73		
	Male	leader	1.80	1.00		b = −0.60, 95% CI = [−0.04, 0.44], SE = 0.22, t(58.00) = −6.01, p < .001
		follower	5.40	0.72		
race	Female	leader	2.28	1.13	b = 0.07, 95% CI = [−0.56, 0.70], SE = 0.32, t(84.00) = 0.21, p = .83	b = −0.34, 95% CI = [−0.85, 0.51], SE = 0.26, t(28.00) = −0.33, p = 0.19
		follower	2.62	1.35		
	Male	leader	1.90	1.08		b = −0.28, 95% CI = [−0.74, 0.47], SE = 0.24, t(28.00) = −0.16, p = 0.26
		follower	2.17	0.93		
**Panel B: Perceived Dominance**						
**Dimension**	**Generated by**	**Condition**	**Mean** ^b^	**SD**	**Interaction Results**	**Regression Results**
competence^c^	Female	leader	4.55	1.12	b = 0.06, 95% CI = [−0.54, 0.66], SE = 0.31, t(90.00) = 0.21, p = .84	b = 0.35, 95% CI = [0.01, 0.35], SE = 0.18, t(30.00) = 2.01, p = 0.05
		follower	4.19	0.95		
	Male	leader	4.58	1.12		b = 0.42, 95% CI = [−0.12, 0.54], SE = 0.28, t(30.00) = 1.51, p = 0.14
		follower	4.16	1.13		
dominance	Female	leader	4.53	0.86	b = 0.13, 95% CI = [−0.55, 0.81], SE = 0.35, t(87.00) = 0.38, p = .70	b = 2.00, 95% CI = [1.54, 0.46], SE = 0.23, t(58.00) = 8.61, p < .001
		follower	2.53	0.94		
	Male	leader	4.57	0.86		b = 2.13, 95% CI = [1.61, 0.53], SE = 0.27, t(58.00) = 7.95, p < .001
		follower	2.43	1.19		
powerfulness	Female	leader	4.27	1.01	b = −0.20, 95% CI = [−0.77, 0.37], SE = 0.29, t(87.00) = −0.69, p = .49	b = 1.47, 95% CI = [1.06, 0.41], SE = 0.21, t(29.00) = 7.07, p < .001
		follower	2.80	1.03		
	Male	leader	4.13	0.97		b = 1.27, 95% CI = [0.89, 0.38], SE = 0.19, t(29.00) = 6.62, p < .001
		follower	2.87	1.04		
**Panel C: Perceived Positivity**						
**Dimension**	**Generated by**	**Condition**	**Mean** ^b^	**SD**	**Interaction Results**	**Regression Results**
attractiveness	Female	leader	4.10	1.27	b = 0.57, 95% CI = [−0.12, 1.26], SE = 0.35, t(87.00) = 1.61, p = .11	b = 0.00, 95% CI = [−0.61, 0.61], SE = 0.31, t(58.00) = 0.00, p > .99
		follower	4.10	1.12		
	Male	leader	4.50	1.20		b = 0.57, 95% CI = [0.13, 1.00], SE = 0.22, t(29.00) = 2.54, p = .02
		follower	3.93	1.23		
happiness	Female	leader	3.84	0.86	b = 1.35, 95% CI = [0.80, 1.90], SE = 0.28, t(120.00) = 4.77, p < .001	b = −0.19, 95% CI = [−0.61, 0.22], SE = 0.21, t(60.00) = −0.92, p = .36
		follower	4.03	0.80		
	Male	leader	3.87	0.67		b = 1.16, 95% CI = [0.79, 1.54], SE = 0.19, t(69.00) = 6.09, p < .001
		follower	2.71	0.82		
likability	Female	leader	3.59	1.09	b = 1.17, 95% CI = [0.56, 1.78], SE = 0.31, t(84.00) = 3.74, p < .001	b = −0.69, 95% CI = [−1.18, −0.20], SE = 0.25, t(28.00) = −0.77, p = .01
		follower	4.28	1.00		
	Male	leader	3.97	0.82		b = 0.48, 95% CI = [0.05, 0.91], SE = 0.22, t(28.00) = 2.20, p = .04
		follower	3.48	1.06		
trustworthiness	Female	leader	3.26	1.32	b = 0.35, 95% CI = [−0.35, 1.05], SE = 0.36, t(90.00) = 0.99, p = .33	b = −1.13, 95% CI = [−0.72, 0.54], SE = 0.30, t(30.00) = −3.77, p < .001
		follower	4.39	1.17		
	Male	leader	3.29	1.22		b = −0.77, 95% CI = [−0.24, 0.30], SE = 0.24, t(30.00) = −3.23, p = .003
		follower	4.06	1.06		
warmth	Female	leader	3.47	1.41	b = 1.23, 95% CI = [0.54, 1.92], SE = 0.35, t(87.00) = 3.48, p < .001	b = −0.67, 95% CI = [−0.15, 0.18], SE = 0.25, t(29.00) = −2.71, p = .01
		follower	4.13	1.14		
	Male	leader	3.83	1.23		b = 0.57, 95% CI = [0.07, 1.06], SE = 0.25, t(29.00) = 2.25, p < .03
		follower	3.27	1.11		

^a^Higher ratings in Gender indicate the image was more likely perceived as Female, and higher ratings in Race indicate the image was more likely perceived as African American.

^b^All ratings are on a 1 − 6 point scale.

For example, 1 means “Definitely MALE/ WHITE AMERICAN” and 6 means “Definitely FEMALE/ AFRICAN AMERICAN” in the perceived demographics ratings.

^c^In the other ratings, 1 means “Definitely INCOMPETENT/SUBMISSIVE/ POWERLESS/ UNATTRACTIVE/ UNHAPPY/ UNLIKABLE/ UNTRUSTWORTHY/ COLD” and 6 means “Definitely COMPETENT/ DOMINANT/ PWERFUL/ ATTRACTIVE/ HAPPY/ LIKABLE/ TRUSTWORTHY/ WARM”.

For happiness ratings, there was a significant interaction (*b* =  1.35, 95% CI [0.80, 1.90], *SE* =  0.28, *t*(120.00) =  4.77, *p* < .001). Similarly, significant interactions emerged for likability (*b* =  1.17, 95% CI [0.56, 1.78], *SE* =  0.31, *t*(84.00) =  3.74, *p* < .001) and warmth (*b* =  1.23, 95% CI [0.54, 1.92], *SE* =  0.35, *t*(87.00) =  3.48, *p* < .001). Simple effects analyses revealed similar patterns across participant gender on these three measures. For perceived happiness, male participant-generated images revealed a significantly positive effect for the leader and follower comparison (*b* =  1.16, 95% CI = [0.79, 1.54], *SE* =  0.19, *t*(69.00) =  6.09, *p* < .001), whereas the effect was no*t* significant for female participant-generated image pairs (*b* =  −0.19, 95% CI = [−0.61, 0.22], *SE* =  0.21, *t*(60.00) =  −0.92, *p* = .36). Furthermore, for the likability and warmth measures, while there were positive differences between the leader and follower image composites for the male participant-generated images (likability: *b* =  0.48, 95% CI = [0.05, 0.91], *SE* =  0.22, *t*(28.00) =  2.20, *p* = .04; warmth: *b* =  0.57, 95% CI = [0.07, 1.06], *SE* =  0.25, *t*(29.00) =  2.25, *p* = .03), there were significant negative differences between the leader and follower image composites for the female participant-generated images (likability: *b* = −0.69, 95% CI = [−1.18, −0.20], *SE* = 0.25, *t*(28.00) = −2.77, *p* = .01; warmth: *b* = −0.67, 95% CI = [−1.15, −0.18], *SE* = 0.25, *t*(29.00) = −2.71, *p* = .012). This contrast between female participant-generated and male participant-generated images suggests that for female participants, their mental representation of a White leader was less positive than a White follower, but for male participants, their mental representation of a White leader was both more able and positive than a follower.

Thus, as with Studies 1–3, relative to imagined followers, people imagined a White leader to look more dominant, competent, and powerful. As with Studies 1 and 2, the mental representation of a leader appeared to be more capable and more positive in the minds of the male participants, but simply more capable in the minds of the female participants. Thus, both Study 2 (constraining faces to be male) and Study 4 (constraining faces to be White) produced images of leaders that look strikingly similar to those produced in Study 1, which began with a race-ambiguous and gender-ambiguous base face.

When beginning with a blank social-categorical slate, the perceptual features that come to mind when people think of leaders (rather than followers) look very similar to those that come to mind when thinking of male leaders and when thinking of White leaders. And male participants, but not female participants, hold the view that this common image of leader looked both competent and positive. When people imagined female leaders, however, different features came to mind for female participants. For female leaders, both female and male participants’ mental representation looked competent and likable.

## Study 5: What does a Black leader look like?

Our final study included target images of Black individuals to test whether people hold yet another set of expectations for Black targets to look like a leader.

### Methods

The procedures of Studies 5a-5c were identical to Studies 2a-4c, with the only difference being that the base image (with visual noise superimposed upon) was Black, but gender-ambiguous; 243 participants completed Study 5a, where participants selected, across 300 trials, which of two images looked more like a leader (*M* = 38.84 years, *SD* =  14.32 years, 120 women, 123 men; 70.37% White, 8.23% Black, 7.82% Hispanic, 8.23% Asian, and 5.35% other racial background participants), and 239 participants completed Study 5b, selecting across the 300 trials which image looks more like a follower (*M* =  40.87 years, *SD* =  14.81 years, 116 women, 123 men; 72.38% White, 10.46% Black, 5.02% Hispanic, 8.79% Asian, and 3.35% other racial background participants). Three participants were 5a, and three participants were excluded in Study 5b, due to the same exclusion criteria as in Studies 1a and 1b.

**Fig 5:**
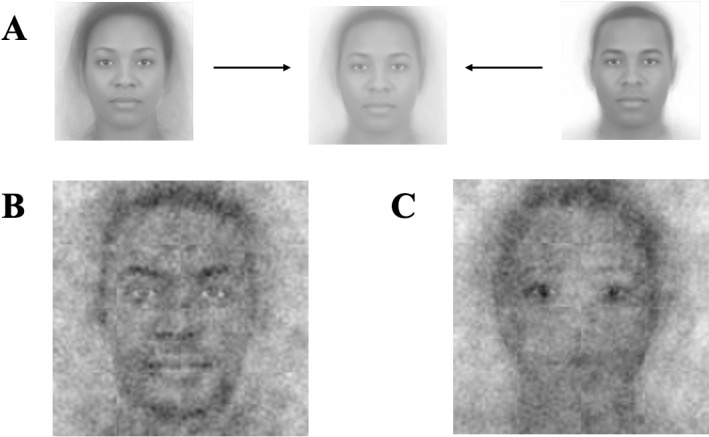
Visual composites for Study 5. Image attribution: “Ma, Correll, & Wittenbrink (2015). The Chicago Face Database: A Free Stimulus Set of Faces and Norming Data. Behavior Research Methods, 47, 1122-1135.” Reprinted from Chicago Face Database under a CC BY license, with permission from The University of Chicago Center for Decision Science, original copyright 2015. (A) Black Composite Image. (B) Visual composite of all leader selections. (C) Visual composite of all follower selections.

In Studies 5a & 5b, the base image was derived from morphing Black female and Black male images to create the Black base image. To ensure the morphed image was truly gender-ambiguous, we pretested several versions of the morphed image and selected the one that participants perceived as most ambiguous with respect to gender (see Fig 5 for the morphed image and SOM for the pretesting procedures).

In Study 5c, again, each participant rated the 26 composite images on one randomly selected dimension of the ten (305 participants, *M* =  38.07 years, *SD* =  12.37 years, 154 women, 151 men; 71.15% White, 10.82% Black, 8.85% Hispanic, 7.87% Asian, and 1.31% other racial background participants), thus yielding about 30 participants per each rating. Three participants were excluded due to the same exclusion criteria as in Study 1c.

### Results and discussion

As in the earlier studies, we again implemented multilevel models, including random intercepts for image and rater.

The Black leader composite images were judged as appearing more male than the Black follower composite images, *b* = −2.58, 95% CI = [−2.70, −2.47], *SE* = 0.06, *t*(762.00) = −43.97, *p* < 0.001. Similar to Study 4c, even though the base image was pre-tested to appear Black, the leader composite images were judged as appearing more Black than the follower composite images, *b* = 0.96, 95% CI = [0.86, 1.07], *SE* = 0.05, *t*(762.00) = 18.12, *p* < 0.001, though the difference on the race dimension was smaller than that on the gender dimension. The descriptive statistics of these measures can be found in Table 9.

**Table 9:** Descriptive statistics for the leader and follower ratings. Study 5c.

Category	Dimension	Condition	Mean^b^	SD
Perceived Demographics	gender^a^	leader	1.78	0.87
		follower	4.37	0.95
	race	leader	5.09	0.90
		follower	4.13	1.06
Perceived Dominance	competence^c^	leader	4.62	1.10
		follower	3.42	1.20
	dominance	leader	4.27	1.00
		follower	2.84	1.08
	powerfulness	leader	4.25	0.90
		follower	2.58	0.86
Perceived Positivity	attractiveness	leader	3.55	1.32
		follower	2.53	1.28
	happiness	leader	3.70	1.00
		follower	3.04	1.08
	likability	leader	3.94	1.17
		follower	3.21	1.10
	trustworthiness	leader	3.61	1.17
		follower	3.08	1.23
	warmth	leader	3.91	1.10
		follower	3.06	1.14

^a^Higher ratings in Gender indicate the image was more likely perceived as Female, and higher ratings in Race indicate the image was more likely perceived as African American.

^b^All ratings are on a 1–6 point scale.

For example, 1 means “Definitely MALE/ WHITE AMERICAN” and 6 means “Definitely FEMALE/ AFRICAN AMERICAN” in the perceived demographics ratings.

^c^In the other ratings, 1 means “Definitely INCOMPETENT/SUBMISSIVE/ POWERLESS/ UNATTRACTIVE/ UNHAPPY/ UNLIKABLE/ UNTRUSTWORTHY/ COLD” and 6 means “Definitely COMPETENT/ DOMINANT/ PWERFUL/ ATTRACTIVE/ HAPPY/ LIKABLE/ TRUSTWORTHY/ WARM”.

Relative to the Black follower composites, the Black leader composites received more favorable ratings for items that represent dominance: more competent (*b* =  1.20, 95% CI =  [1.08, 1.32], *SE* =  0.06, *t*(762.00) =  19.20, *p* <  0.001), more dominant (*b* =  1.42, 95% CI =  [1.30, 1.55], *SE* =  0.06, *t*(712.00) =  22.41, *p* <  0.001), and more powerful (*b* =  1.67, 95% CI =  [1.57, 1.77], *SE* =  0.05, *t*(787.00) =  32.72, *p* <  0.001).

At the same time, relative to the Black follower composites, the Black leader composites also received more favorable ratings for items that represent positivity: more attractive (*b* =  1.02, 95% CI =  [0.90, 1.14], *SE* =  0.06, *t*(737.00) =  16.36, *p* <  0.001), happier (*b* =  0.65, 95% CI =  [0.53, 0.78], *SE* =  0.06, *t*(762.00) =  10.70, *p* <  0.001), more likable (*b* =  0.73, 95% CI =  [0.61, 0.85], *SE* =  0.06, *t*(762. 00) =  11.94, *p* <  0.001), more trustworthy (*b* =  0.53, 95% CI =  [0.39, 0.66], *SE* =  0.07, *t*(712.00) =  7.76, *p* <  0.001), and more warm (*b* =  0.85, 95% CI =  [0.72, 0.99], *SE* =  0.07, *t*(737.00) =  12.44, *p* <  0.001). Again, the magnitude of the effects on the positivity dimensions are relatively small compared to the ability dimensions (i.e., comparing *b*s and their confidence intervals), as in earlier studies.

The interaction analyses between the gender of the image-generating participants and the leader/follower status revealed several significant effects on the ability and positivity dimensions (See Table 10). On the ability dimension, there was a significant interaction on perceived competence (*b* =  0.81, 95% CI = [0.15, 1.47], *SE* =  0.33, *t*(90.00) =  2.42, *p* = .02). While both female and male participant-generated leader images were rated more competent than the follower images, this difference was larger in male participant-generated images than female participant-generated images (male: *b* =  2.06, 95% CI = [1.54, 2.58], *SE* =  0.27, *t*(60.00) =  7.79, *p* < .001; female: *b* =  1.26, 95% CI = [0.87, 1.64], *SE* =  0.20, *t*(30.00) =  6.40, *p* < .001).

**Table 10:** Descriptive statistics and regression results for the female and male participant-generated leader and follower ratings. Study 5c.

Panel A: Perceived Demographics						
Dimension	Generated by	Condition	Mean^b^	SD	Interaction Results	Regression Results
gender^a^	Female	leader	1.84	1.07	b = −0.23, 95% CI = [−0.81, 0.35], SE = 0.30, t(90.00) = −0.76, p = .45	b = −0.71, 95% CI = [−0.15, −0.26], SE = 0.23, t(60.00) = − 1.94, p < .001
		follower	4.55	0.68		
	Male	leader	1.45	0.62		b = −0.94, 95% CI = [−0.34, −0.53], SE = 0.20, t(60.00) = −4.33, p < .001
		follower	4.39	0.95		
race	Female	leader	5.03	0.91	b = −0.13, 95% CI = [−0.68, 0.42], SE = 0.28, t(90.00) = −0.46, p = .65	b = 1.10, 95% CI = [0.69, 1.51], SE = 0.21, t(30.00) = 5.24, p < .001
		follower	3.94	1.03		
	Male	leader	5.03	1.02		b = 0.97, 95% CI = [0.51, 1.43], SE = 0.23, t(30.00) = 4.13, p < .001
		follower	4.06	1.18		
**Panel B: Perceived Dominance**						
**Dimension**	**Generated by**	**Condition**	**Mean** ^b^	**SD**	**Interaction Results**	**Regression Results**
competence^c^	Female	leader	4.65	1.02	b = 0.81, 95% CI = [0.15, 1.47], SE = 0.33, t(90.00) = 2.42, p = .02	b = 1.26, 95% CI = [0.87, 1.64], SE = 0.20, t(30.00) = 6.40, p < .001
		follower	3.39	1.17		
	Male	leader	4.94	0.96		b = 2.06, 95% CI = [1.54, 2.58], SE = 0.27, t(60.00) = 7.79, p < .001
		follower	2.87	1.12		
dominance	Female	leader	4.17	1.14	b = 0.24, 95% CI = [−0.53, 1.01], SE = 0.40, t(84.00) = 0.61, p = .54	b = 1.38, 95% CI = [0.89, 1.87], SE = 0.25, t(28.00) = 5.51, p < .001
		follower	2.79	1.01		
	Male	leader	4.55	0.91		b = 1.62, 95% CI = [1.02, 2.22], SE = 0.30, t(56.00) = 5.33, p < .001
		follower	2.93	1.36		
powerfulness	Female	leader	4.13	0.87	b = 0.34, 95% CI = [−0.21, 0.89], SE = 0.28, t(93.00) = 1.21, p = .23	b = 1.78, 95% CI = [1.43, 2.13], SE = 0.18, t(31.00) = 10.00, p < .001
		follower	2.34	0.79		
	Male	leader	4.50	1.08		b = 2.13, 95% CI = [1.62, 2.63], SE = 0.26, t(37.20) = 8.27, p < .001
		follower	2.38	0.98		
**Panel C: Perceived Positivity**						
**Dimension**	**Generated by**	**Condition**	**Mean** ^b^	**SD**	**Interaction Results**	**Regression Results**
attractiveness	Female	leader	3.63	1.38	b = 0.90, 95% CI = [0.22, 1.58], SE = 0.35, t(87.00) = 2.58, p = .01	b = 0.73, 95% CI = [0.20, 1.26], SE = 0.27, t(29.00) = 2.71, p = .01
		follower	2.90	1.47		
	Male	leader	4.07	1.36		b = 1.63, 95% CI = [1.09, 2.18], SE = 0.28, t(29.00) = 5.89, p < .001
		follower	2.43	1.33		
happiness	Female	leader	4.29	0.86	b = 0.03, 95% CI = [−0.55, 0.61], SE = 0.30, t(90.00) = 0.11, p = .91	b = 0.77, 95% CI = [0.31, 1.23], SE = 0.24, t(30.00) = 3.29, p = .002
		follower	3.52	1.18		
	Male	leader	3.65	0.80		b = 0.81, 95% CI = [0.39, 1.23], SE = 0.21, t(30.00) = 3.76, p < .001
		follower	2.84	1.00		
likability	Female	leader	4.10	1.16	b = 0.55, 95% CI = [−0.17, 1.27], SE = 0.36, t(90.00) = 1.51, p = .14	b = 0.77, 95% CI = [0.26, 1.29], SE = 0.26, t(30.00) = 2.97, p = .005
		follower	3.32	1.17		
	Male	leader	4.19	1.17		b = 1.32, 95% CI = [0.76, 1.88], SE = 0.29, t(60.00) = 4.62, p < .001
		follower	2.87	1.09		
trustworthiness	Female	leader	4.03	1.05	b = 0.24, 95% CI = [−0.53, 1.01], SE = 0.39, t(84.00) = 0.61, p = .54	b = 0.69, 95% CI = [0.17, 1.21], SE = 0.27, t(28.00) = 2.58, p = .02
		follower	3.34	1.42		
	Male	leader	3.83	1.23		b = 0.93, 95% CI = [0.29, 1.57], SE = 0.33, t(56.00) = 2.85, p = .006
		follower	2.90	1.26		
warmth	Female	leader	4.43	1.19	b = 0.27, 95% CI = [−0.47, 1.01], SE = 0.38, t(87.00) = 0.71, p = .48	b = 1.00, 95% CI = [0.50, 1.50], SE = 0.25, t(29.00) = 3.94, p < .001
		follower	3.43	1.22		
	Male	leader	3.97	1.19		b = 1.27, 95% CI = [0.74, 1.80], SE = 0.27, t(29.00) = 4.68, p < .001
		follower	2.70	1.09		

^a^Higher ratings in Gender indicate the image was more likely perceived as Female, and higher ratings in Race indicate the image was more likely perceived as African American.

^b^All ratings are on a 1-6 point scale.

For example, 1 means “Definitely MALE/ WHITE AMERICAN” and 6 means “Definitely FEMALE/ AFRICAN AMERICAN” in the perceived demographics ratings.

^c^In the other ratings, 1 means “Definitely INCOMPETENT/SUBMISSIVE/ POWERLESS/ UNATTRACTIVE/ UNHAPPY/ UNLIKABLE/ UNTRUSTWORTHY/ COLD” and 6 means “Definitely COMPETENT/ DOMINANT/ PWERFUL/ ATTRACTIVE/ HAPPY/ LIKABLE/ TRUSTWORTHY/ WARM”.

On the positivity dimensions, a significant interaction was found on perceived attractiveness (*b* =  0.90, 95% CI [0.22, 1.58], *SE* =  0.35, *t*(87.00) =  2.58, *p* = .01). While both female and male participant-generated leader images were rated more attractive than the follower images, this difference was larger in male participant-generated images than female participant-generated images (male: *b* =  1.63, 95% CI [1.09, 2.18], *SE* =  0.28, *t*(29.00) =  5.89, *p* < .001; female: *b* =  0.73, 95% CI [0.20, 1.26], *SE* =  0.27, *t*(29.00) =  2.71, *p* < .001). No interaction effects were found in the other measures (see Table 10). These results showed a different pattern than those from Studies 1, 2 (male leaders), and 4 (White leaders), but were somewhat similar to those from Study 3 (female leaders). Both the female and male participant-generated Black leader images were rated higher on the competence and positivity dimensions than the follower images. These results suggest that for people’s mental representations of Black leaders, both female participants and male participants believe that Black leaders need to look both capable and positive.

Thus, as with Studies 1-4, relative to imagined followers, people imagined a *Black* leader to look more dominant, competent, and powerful. And as in Study 3 (faces constrained to be female), relative to imagined followers, people imagined Black leaders to look more positive than Black followers.

## General discussion

Across all studies—when imagining leaders, male leaders, female leaders, White leaders and Black leaders—our participants imagined a face that looked more dominant, competent, powerful, and attractive, compared to the imagined faces of followers. These studies reveal that people have perceptual expectations for how leaders should look, such that when faces did not appear dominant, competent, powerful, and attractive, those faces looked more like followers than leaders.

Simultaneously, our studies revealed clear gender and racial biases in perceptual expectations for leaders. At default, when people are simply asked to imagine a leader, relative to a follower, they imagine someone who looks like a White male. These studies offer evidence for a perceptual demand-side explanation for the underrepresentation of women and minorities in top leadership positions. This *perceptual imprinting* of leaders as largely male and White is reflected in people’s visual templates of leaders’ faces, showcasing a perceptual leadership bias. Presumably, these mental representations of leaders and followers become skewed over time, given long-term societal exposure to a disproportionate number of White males in top-level positions, resulting in a reinforced cognitive association between leadership and a specific, narrow appearance—namely, that of a White male.

Critically, we also demonstrated that the perceptual features people expect in leaders’ faces vary according to race and gender. When participants generated images of Black leaders’ faces, they needed to appear more positive (likability), whereas White leaders’ faces did not have to look likable in everyone’s minds (only appearing more likeable in men’s eyes), and were even overall allowed to look less trustworthy than followers (and less likable in females’ eyes). For Black leaders, positive perceptions likely serve to offset racial bias [23], whereas White leaders may more easily be able to use dominance to access leadership positions [34]. Our findings suggest that people internalize disparate expectations for White and Black leaders, which in turn affects how they envision faces of leaders to look depending on race.

Across all studies, when people imagined the face of a leader, the resulting faces looked more dominant, competent, and powerful than imagined followers, and this included female leaders. Thus, to look like a leader, one must look commanding and capable. Problematically, these traits are conceptually overlapping with notions of masculinity [22] and facial appearances of dominance are also perceived as making a face look masculine [21]. And thus these traits, while overlapping with conceptions of leadership, are often linked with perceptions of masculinity. Additionally, leaders were consistently judged as more attractive than followers across all five studies. This finding aligns with recent research suggesting that perceived attractiveness is often associated with perceived competence [35].

What does this mean for female leaders? How are female leaders mentally represented given that leadership is highly associated with masculinity? When imagining the face of female leaders, our participants envisioned faces that appeared more dominant, competent, and powerful than imagined female followers. However, participants also expected female leaders to appear more female and likable, regardless of whether the images came from male participants or female participants. Female participants also expected female leaders to appear happy. In contrast, in the male leader study, female participants did not imagine male leaders as looking more happy than male followers, whereas male participants did imagine male leaders as looking more happy than male followers.

Thus, it seems that to offset negative perceptions of a female leader who appears dominant, she must also look likable and happy. This is reminiscent of research showing that people expect women to smile more than men due to expectations of affiliativeness [21,36]. Here we show this requirement extends even to perceptual expectations for how female leaders should look - female leaders needed to look more dominant *and* positive to appear leader-like. This finding dovetails with work showing that to be perceived as effective leaders, women need to be judged as able and positive [33] (see also [37]).

In a similar fashion, imagined Black leaders and imagined female leaders both looked more likeable than imagined followers. Therefore, in addition to appearing dominant, competent, and powerful, to look leader-like both Black and female targets needed to appear likeable. This requirement did not extend to imagined male leaders or White leaders across all participants.

The findings on imagined Black leaders align with the proposed benefit of “disarming” cues for Black men. For example, Black chief executive officers (CEOs) have been found to be more babyfaced than White CEOs [23]. That is, to the extent Black men are perceived as threatening due to societal stereotypes, positive appearances would offset this perceived threat. In parallel, we found that for an imagined Black target to look like a leader, the face needed to appear likeable.

Notably, previous studies using a similar reverse-correlation paradigm to examine topics such as welfare and social inequality often find larger race (relative to gender) effects [29]. The fact that we observed a larger gender (relative to race) effect in Study 1 (with leaders looking particularly more male than female) suggests that leadership is a unique domain where implicit gender bias is particularly prominent. Importantly, these results were found within the context of the U.S. and our participant sample consisted of mostly White Americans. The *perceptual imprinting* of leaders in other countries (where the racial breakdown of the country’s population differs) would likely be different. For instance, countries with different cultural representations of leadership might show distinct patterns in how people mentally visualize their leaders. Furthermore, the mental representation of leaders of African American, Asian American and other minority groups within the U.S. might differ from the current findings given well-documented other-race effects in facial perception, where people show better recognition and memories of faces from their own racial group [38]. Therefore, future research would benefit from examining how these mental representations of leaders vary across different cultural contexts and national groups.

At the highest levels of leadership, gender and racial biases are vastly pronounced. Our findings charter new territory by documenting the perceptual correlates of one of the most highly studied demand-side explanations—gender bias and race bias. In so doing, we demonstrate a pernicious side-effect of the status quo, whereby gender and racial imbalance in top positions pervades people’s basic perceptual representations of leaders.

In sum, when considering the face of a leader, relative to a follower, people tend to be biased toward thinking of a White male—and for female and minority leaders, people have different perceptual standards, as expressed by different expectations for what female leaders and minority leaders look like. If the trend of having predominantly White male leaders in society continues, this perceptual imprinting of bias may be more and more difficult to reverse.

## Supporting information

S1 File Supplemental online materials. This file includes the pre-test procedures for identifying ambiguous images and other descriptive statistical results.
(DOCX)
