# Supplementary material for: The look of a leader
Source: PLoS One. 2025 Apr 3;20(4):e0320836. doi: 10.1371/journal.pone.0320836 (PMC11967962; doi:10.1371/journal.pone.0320836)
Supplement: S1 File — This file includes the pre-test procedures for identifying ambiguous images and other descriptive statistical results. (DOCX) [file pone.0320836.s001.docx]

# **Supporting Information**

## **Pretesting the Morphed Images**

Certain demographic categories (e.g., Black, female) are closely associated with other social categories [1]. In order to achieve the goal of having a gender-ambiguous Black or White base image and a race-ambiguous female or male image, we pretested a set of images through morphing 2 or 4 ambiguous mono-social categorical images from the Chicago Face Dataset [2] with different proportions using the software FantaMorph and present the results here.

**Participants and Method**

We recruited two hundred participants on CloudResearch Connect and received 200 responses (*M* = 35.69 years, *SD* = 10.49 years, 98 women and 102 men). Each participant went through the images from Figure 1 one by one and answered the question, “how MALE or FEMALE does this person look?” Next the same set of participants went through the images one more time, answering the question, “how much of a WHITE-AMERICAN or AFRICAN-AMERICAN does this person look like?” Both questions were on six-point scales ranging from, (1) Definitely Male to (6) Definitely Female (6) for the gender question and (1) Definitely White American to (6) Definitely African American (6) for the race question.

**Results**

We compared the averages to the midpoint of the scale, the value at which the participants deemed the image in between the two endpoints. From each category, we kept the image with an average rating that was closest to 3.5. The results are presented in Table S1 and the selected images (boxed) are presented in Figure S1.

| **Table S1. Base Image Selection Results** | | | | | | | |
| --- | --- | --- | --- | --- | --- | --- | --- |
| Ambiguous Base Image | 20% Black Female/ Male, 30% White Female/ Male | 20% Black/ White Female, 30% Black/ White Male | **25% Black/ White Female/ Male** | 30% Black/ White Female, 20% Black/ White Male | 30% Black Female/ Male, 20% White Female/ Male |  |  |
| (S1) |  |  |  |  |  |  |  |
| Race Ratings | M = 3.34 | M = 2.82 | **M = 3.40** | M = 3.86 | M = 3.63 |  |  |
|  | SD = 1.15 | SD = 1.12 | **SD = 1.27** | SD = 1.17 | SD = 1.24 |  |  |
| Gender Ratings | M = 2.88 | M = 3.41 | **M = 3.62** | M = 4.23 | M = 4.18 |  |  |
|  | SD = 1.05 | SD = 1.01 | **SD = 1.11** | SD = 0.94 | SD = 0.95 |  |  |
| Male Base Image (S2) | 35% | 40% | 45% | 50% | **55%** | 60% | 65% Black Image Blend |
|  | M = 2.68 | M = 3.02 | **M = 3.44** | M = 3.78 | M = 4.18 | M = 4.46 | M = 4.81 |
| Race Ratings | SD = 1.11 | SD = 1.22 | **SD = 1.12** | SD = 1.11 | SD = 0.99 | SD = 1.09 | SD = 1.00 |
| Female Base Image (S3) | 35% | 40% | 45% | 50% | **55%** | 60% | 65% Black Image Blend |
|  | M = 2.44 | M = 2.87 | M = 3.08 | M = 3.29 | **M = 3.62** | M = 3.90 | M = 4.25 |
| Race Ratings | SD = 1.1 | SD = 1.15 | SD = 1.17 | SD = 1.03 | **SD = 1.11** | SD = 1.07 | SD = 1.08 |
| White Base Image (S4) | 35% | 40% | 45% | **50%** | 55% | 60% | 65% Female Image Blend |
|  | M = 2.47 | M = 2.76 | M = 3.29 | **M = 3.63** | M = 4.07 | M = 4.32 | M = 4.62 |
| Gender Ratings | SD = 1.16 | SD = 1.22 | SD = 1.19 | **SD = 1.28** | SD = 1.31 | SD = 1.28 | SD = 1.18 |
| Black Base Image (S5) | 35% | 40% | 45% | **50%** | 55% | 60% | 65% Female Image Blend |
|  | M = 2.39 | M = 2.82 | M = 3.21 | **M = 3.58** | M = 4.07 | M = 4.34 | M = 4.69 |
| Gender Ratings | SD = 1.28 | SD = 1.46 | SD = 1.48 | **SD = 1.46** | SD = 1.32 | SD = 1.26 | SD = 1.23 |
| ^a^Higher ratings in Gender indicate the image was more likely perceived as Female, and higher ratings in Race indicate the image was more likely perceived as African American. | | | | | | | |

**Fig S1. Base Image Selection Results
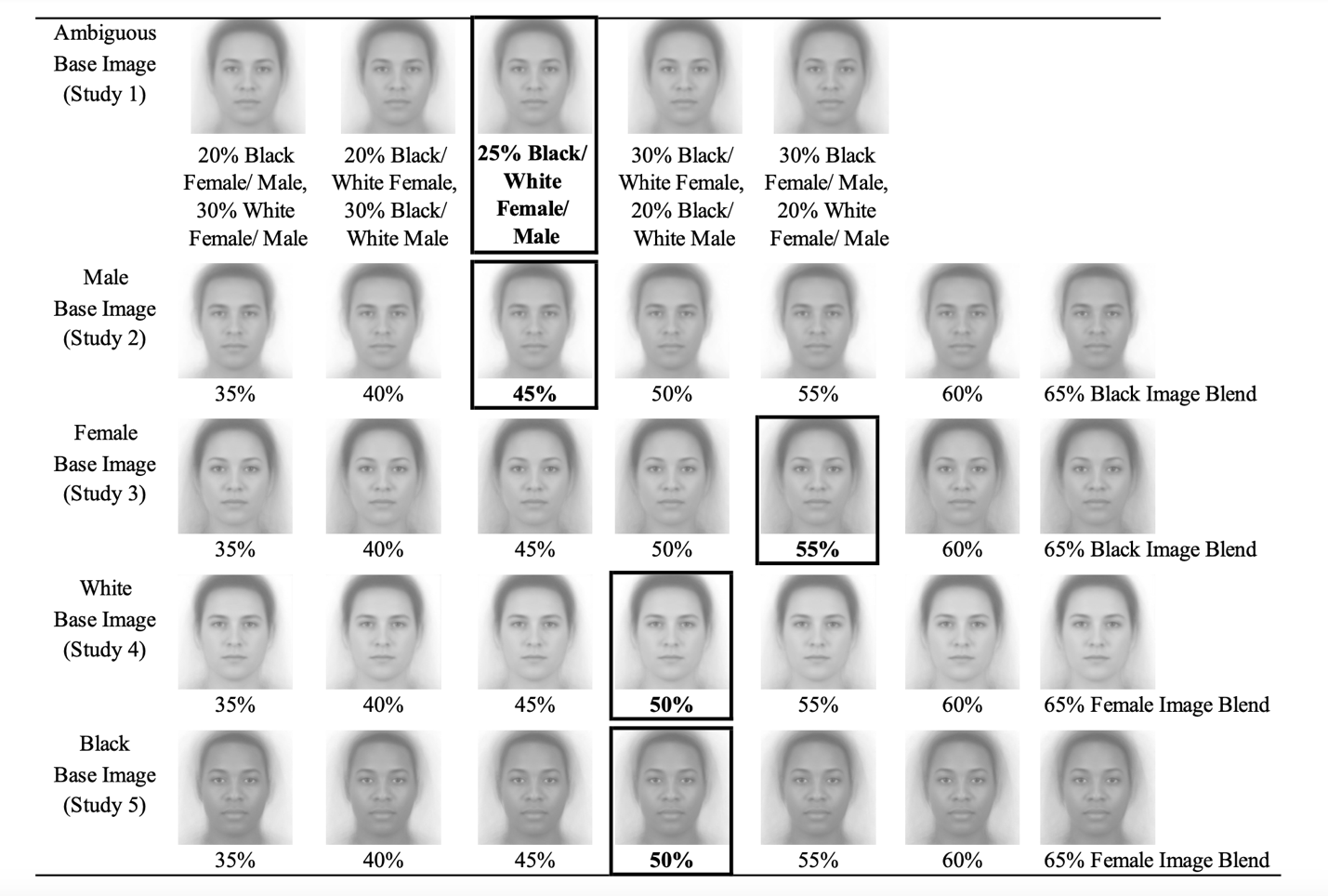
**

**Fig S1. Selected Base Images from Studies 1 - 5. Image attribution: "Ma, Correll, & Wittenbrink (2015). The Chicago Face Database: A Free Stimulus Set of Faces and Norming Data. Behavior Research Methods, 47, 1122-1135." Reprinted from Chicago Face Database under a CC BY license, with permission from The University of Chicago Center for Decision Science, original copyright 2015.**


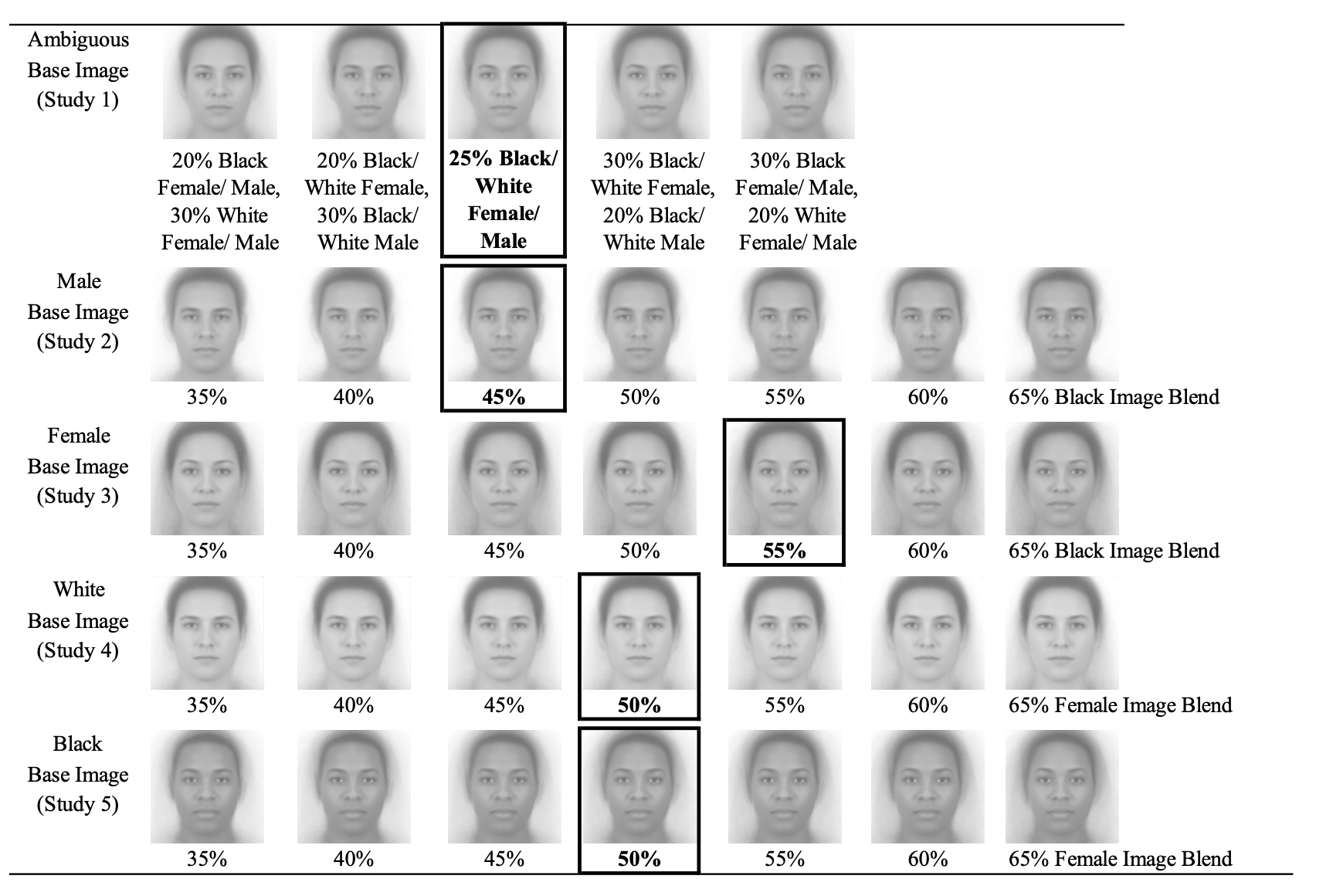


| **Table S2. R-square values for Studies 1-5 from the multilevel linear regression: Dimension ~ cond + (1\|ResponseId) + (1\|image).** | | | | | | | | | | |
| --- | --- | --- | --- | --- | --- | --- | --- | --- | --- | --- |
|  | **Study 1** | | **Study 2** | | **Study 3** | | **Study 4** | | **Study 5** | |
| **Dimension** | R^2^ marginal | R^2^ conditional | R^2^ marg. | R^2^ cond. | R^2^ marg. | R^2^ cond. | R^2^ marg. | R^2^ cond. | R^2^ marg. | R^2^ cond. |
| **gender** | 0.63 | 0.71 | 0.79 | 0.82 | 0.43 | 0.62 | 0.54 | 0.67 | 0.67 | 0.72 |
| **race** | 0.21 | 0.41 | 0.31 | 0.47 | 0.07 | 0.18 | 0.05 | 0.50 | 0.19 | 0.53 |
| **competence** | 0.15 | 0.50 | 0.13 | 0.49 | 0.21 | 0.65 | 0.06 | 0.49 | 0.21 | 0.54 |
| **dominance** | 0.31 | 0.43 | 0.51 | 0.66 | 0.21 | NA^a^ | 0.42 | 0.49 | 0.32 | 0.53 |
| **powerfulness** | 0.44 | 0.52 | 0.49 | 0.57 | 0.38 | 0.53 | 0.30 | 0.61 | 0.47 | 0.63 |
| **attractiveness** | 0.21 | 0.60 | 0.36 | 0.58 | 0.39 | 0.59 | 0.05 | 0.41 | 0.13 | 0.62 |
| **happiness** | 0.00 | 0.34 | 0.16 | 0.43 | 0.09 | 0.42 | 0.04 | 0.30 | 0.09 | 0.38 |
| **likability** | 0.02 | 0.53 | 0.04 | 0.45 | 0.17 | 0.45 | 0.00 | 0.31 | 0.09 | 0.47 |
| **trustworthiness** | 0.01 | 0.20 | 0.01 | 0.41 | 0.06 | 0.32 | 0.04 | 0.46 | 0.05 | 0.43 |
| **warmth** | 0.00 | 0.36 | 0.02 | 0.31 | 0.02 | 0.27 | 0.00 | 0.36 | 0.13 | 0.37 |
| ^a^ NA value is due to singularity issues. | | | | | | | | | | |

**Fig S2. Study 1 Average Values with 95% Confidence Intervals
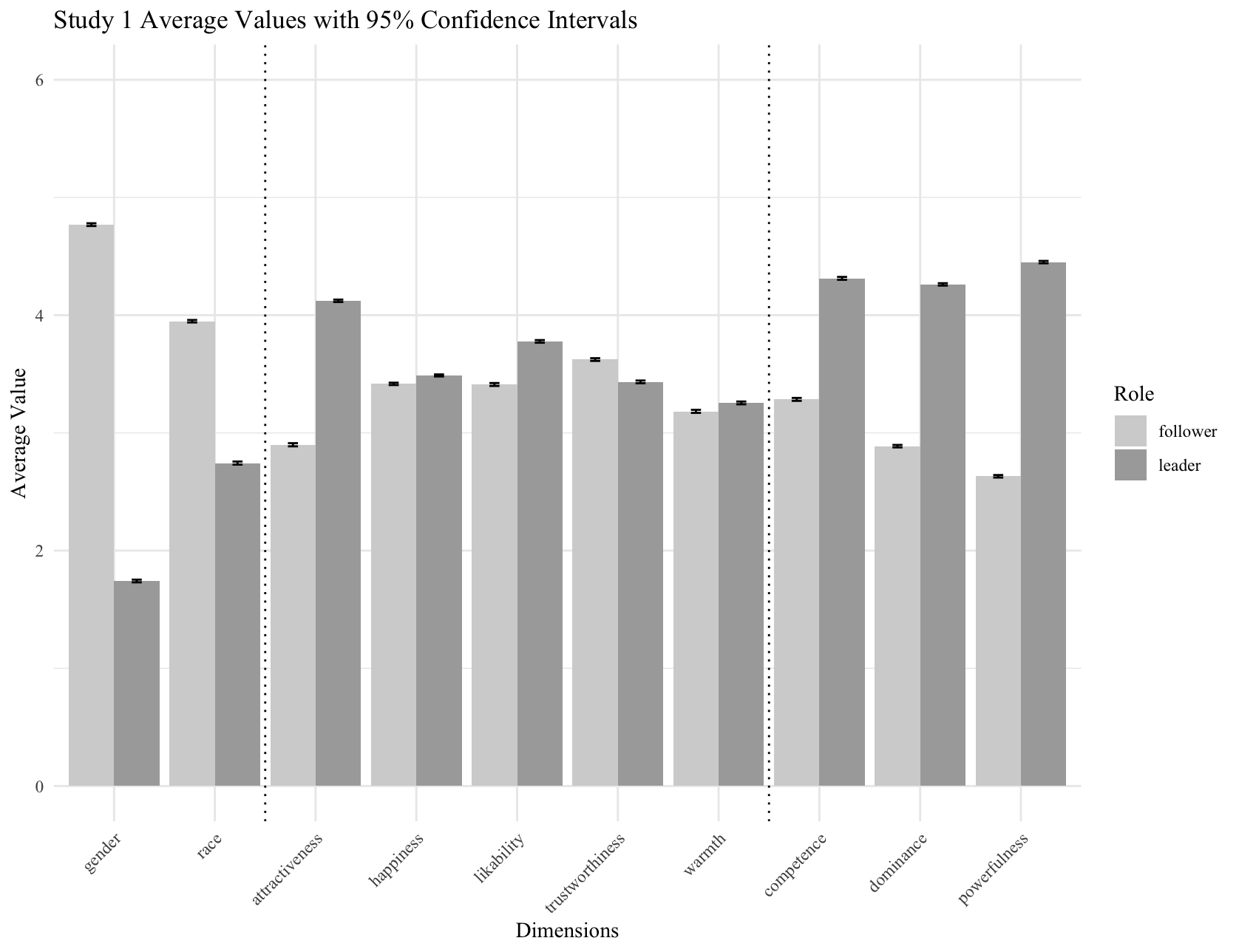
**

**Fig S3. Study 2 Average Values with 95% Confidence Intervals
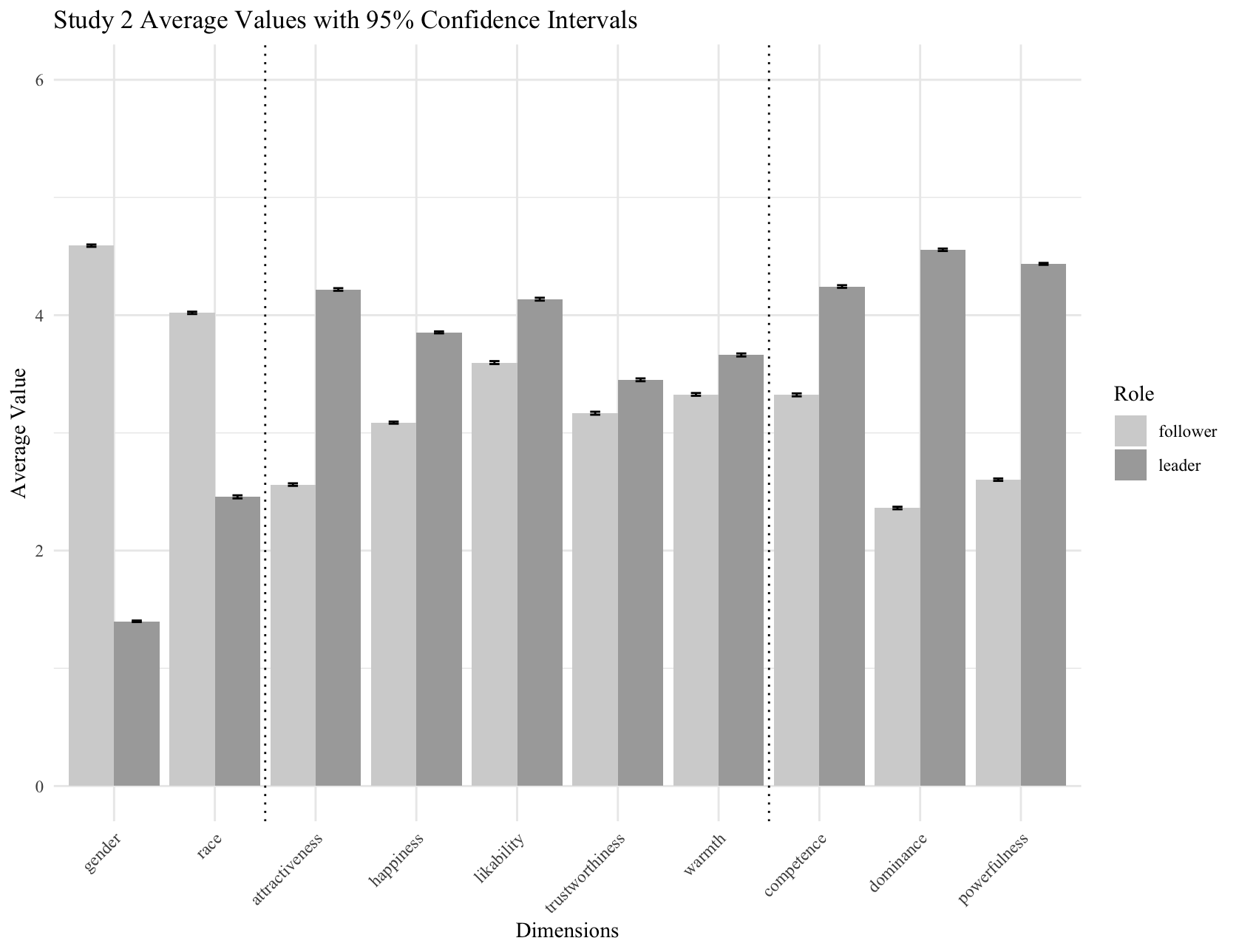
**

**Fig S4. Study 3 Average Values with 95% Confidence Intervals
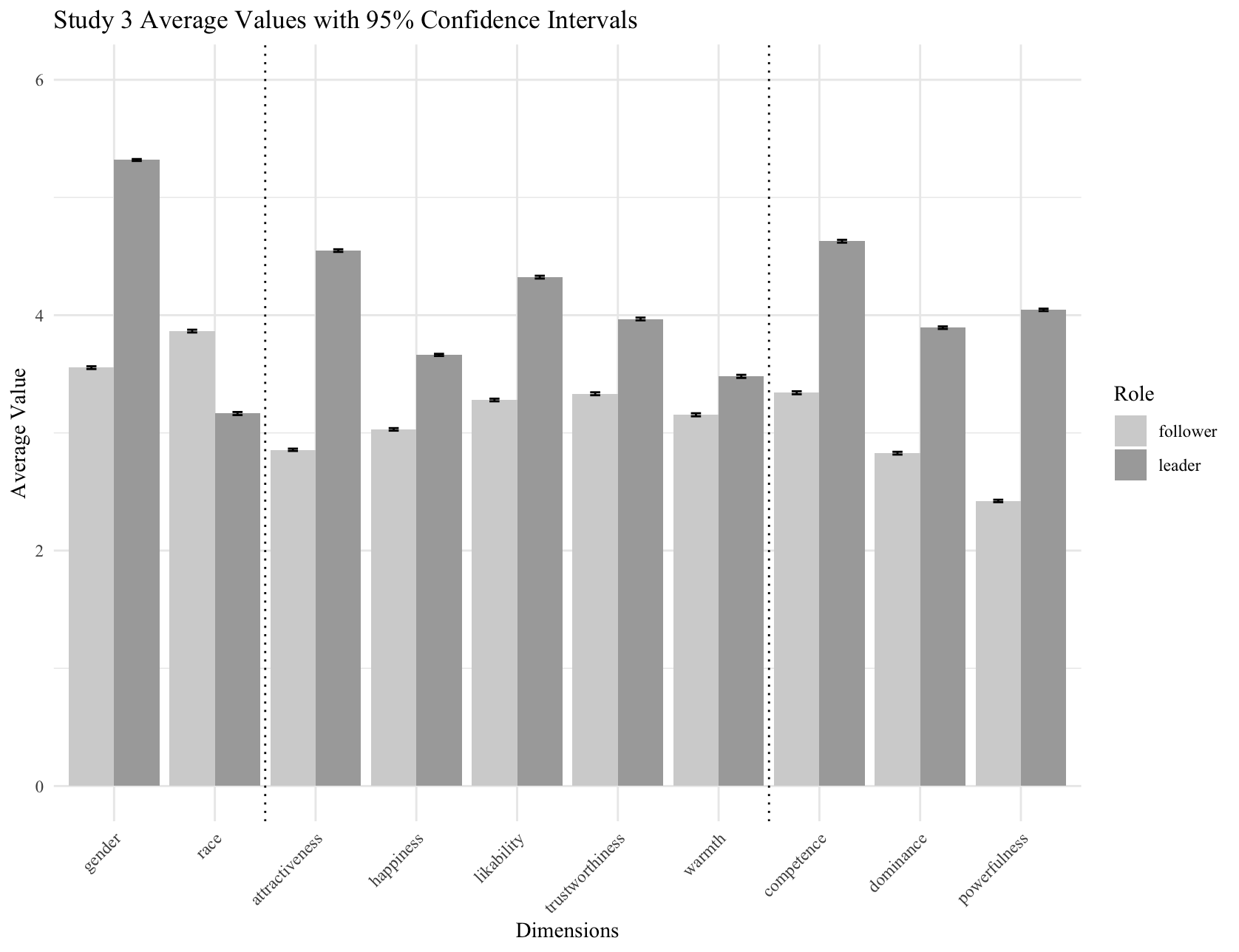
**

**Fig S5. Study 4 Average Values with 95% Confidence Intervals
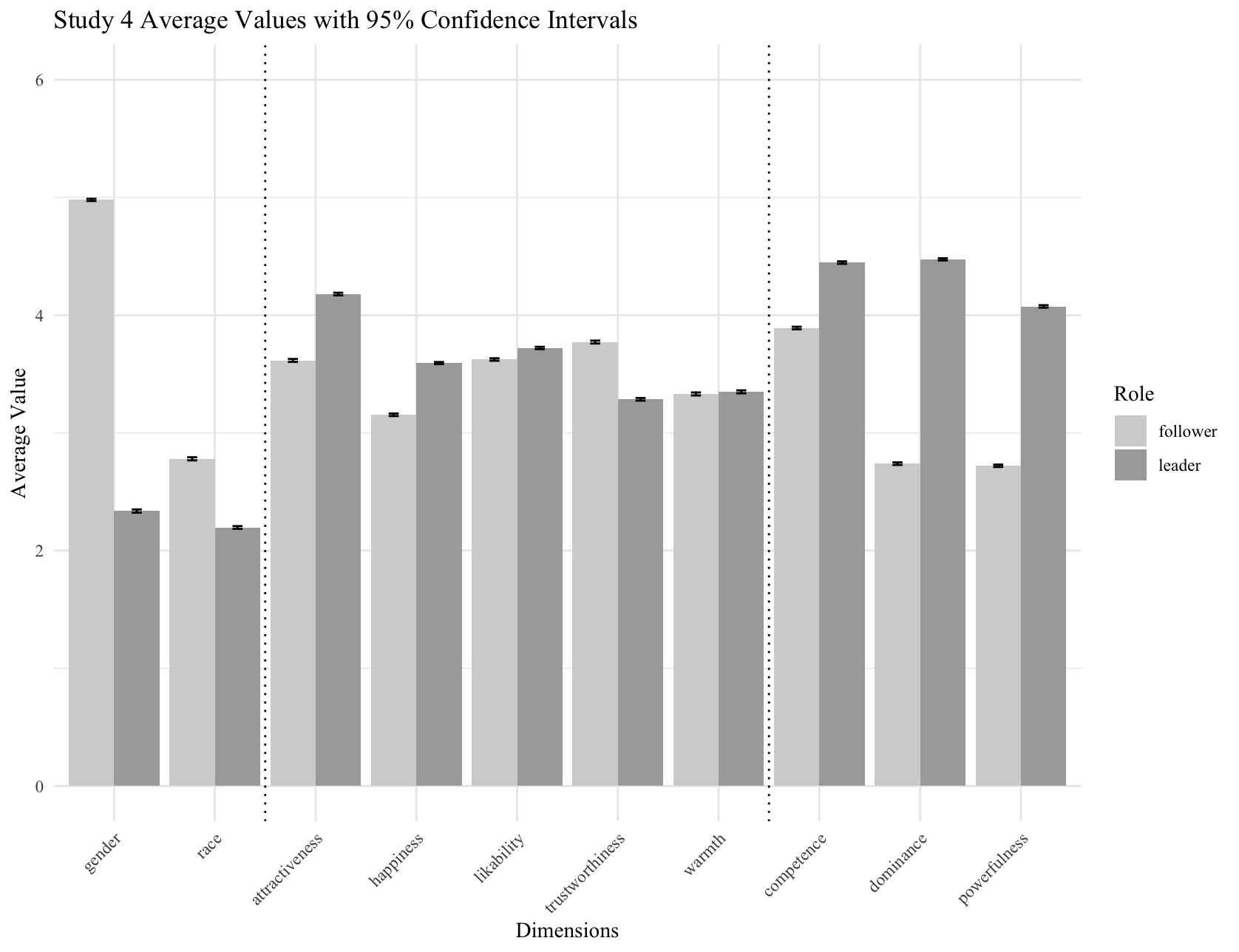
**

**Fig S6. Study 5 Average Values with 95% Confidence Intervals
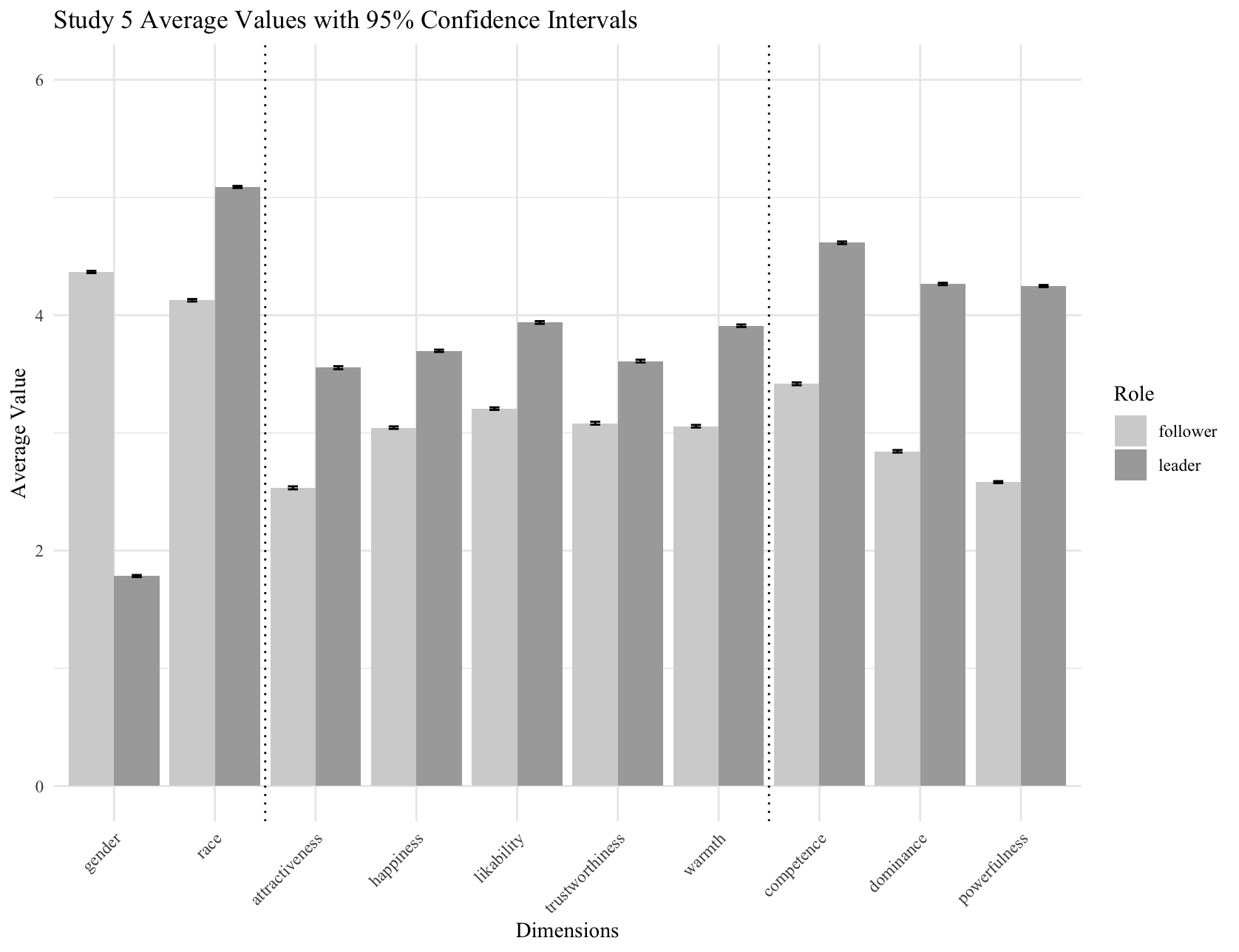
**

## **Supporting Information Reference**

1. Hall EV, Hall AV, Galinsky AD, Phillips KW. MOSAIC: A model of stereotyping through associated and intersectional categories. Academy of Management Review. 2019 Jul;44(3):643-72
2. Ma DS, Correll J, Wittenbrink B. The Chicago face database: A free stimulus set of faces and norming data. Behavior Research Methods. 2015 Jan 13;47(4):1122–35.
